# Rv3091, An Extracellular Patatin-Like Phospholipase in *Mycobacterium tuberculosis*, Prolongs Intracellular Survival of Recombinant *Mycolicibacterium smegmatis* by Mediating Phagosomal Escape

**DOI:** 10.3389/fmicb.2020.532371

**Published:** 2020-09-11

**Authors:** Ziyin Cui, Guanghui Dang, Ningning Song, Yingying Cui, Zhe Li, Xinxin Zang, Hongxiu Liu, Zhongxing Wang, Siguo Liu

**Affiliations:** State Key Laboratory of Veterinary Biotechnology, Division of Bacterial Diseases, Harbin Veterinary Research Institute, Chinese Academy of Agricultural Sciences, Harbin, China

**Keywords:** patatin-like phospholipase, lipase, *Mycobacterium tuberculosis*, phagosome escape, intracellular survival

## Abstract

Patatin-like phospholipases (PLPs) are important virulence factors of many pathogens. However, there are no prevailing studies regarding PLPs as a virulence factor of *Mycobacterium tuberculosis* (Mtb). Analysis of Rv3091, a putative protein of Mtb, shows that it belongs to the PLPs family. Here, we cloned and expressed the *rv3091* gene in *Mycobacterium smegmatis* and, subsequently, conducted protein purification and characterization. We show that it possesses phospholipase A_1_, phospholipase A_2_, and lipase activity. We confirm the putative active site residues, namely, Ser214 and Asp407, using site directed mutagenesis. The Rv3091 is an extracellular protein that alters the colony morphology of *M. smegmatis*. The presence of Rv3091 enhances the intracellular survival capability of *M. smegmatis* in murine peritoneal macrophages. Additionally, it promotes *M. smegmatis* phagosomal escape from macrophages. Moreover, Rv3091 significantly increased the survival of *M. smegmatis* and aggravated lesions in C57BL/6 J murine lungs *in vivo*. Taken together, our results indicate that Rv3091 as an extracellular PLP that is critical to the pathogenicity of mycobacterium as it allows mycobacterium to utilize phospholipids for its growth and provides resistance to phagosome killing, resulting in its enhanced intracellular survival.

## Introduction

Tuberculosis (TB) is a severe consumptive disease that is caused by infection with *Mycobacterium tuberculosis* (Mtb). Mtb is transmitted in humans *via* tiny respiratory droplets generated by coughing or sneezing. In 2018, approximately 10 million (range: 9.0–11.1 million) new TB cases were diagnosed, and 1.2 million HIV-negative individuals (range: 1.1–1.3 million) have died due to TB (World Health Organization, 2019). Despite the maturity of TB prevention and treatment programs over an extended period of time, the disease still has not been completely eradicated (Colditz et al., 1994). The main reason is that Mtb is an intracellular pathogen with a complex structure, a unique secretory system, and various immune evasion mechanisms (Gupta et al., 2012; Stoop et al., 2012; Kallenius et al., 2016). Mtb mainly occurs in macrophages, where they survive and replicate without being killed (Dey and Bishai, 2014). Its intracellular survival plays an essential role in the pathogenesis of Mtb; however, the underlying mechanisms and specific factors remain unclear (Li et al., 2019).

Lipids and the enzymes associated with lipid synthesis and catabolism (i.e., esterases, lipases, and phospholipases) are very important to the Mtb life cycle (Cotes et al., 2008; Singh et al., 2010; Wong et al., 2013). Mtb harbors an unusual cell envelope that exhibits a specific architecture consisting of lipids (phosphatidyl glycerol, cardiolipid, phosphatidyl ethanolamine, phosphatidylinositol, trehalose monomycolate, and trehalose mycolates; Daffe and Marrakchi, 2019). Approximately 8% of the Mtb genome encodes proteins that are associated with lipid metabolism, and lipids comprise up to 40–60% of cell dry weight, whereas it accounts for only 5–10% in other bacterial species (Cole et al., 1998; Daffe and Draper, 1998; Camus et al., 2002; Dedieu et al., 2013; Daffe, 2015). Lipids and their metabolites have been shown to play various roles related to the viability and virulence of Mtb, especially signaling molecules that mediate immune modulation, as well as responses to environmental stimuli for intracellular survival. Therefore, to elucidate the pathogenic mechanisms of Mtb, efforts have been made to develop novel vaccines and drug targets by investigating the structure, function, synthesis, and metabolic pathways of these complex lipids (Banerjee et al., 1994; Vilcheze et al., 2006; North et al., 2014).

As lipids are essential to Mtb viability and pathogenicity, the enzymes related to lipid metabolism are regarded as critical virulence factors in this species (Vromman and Subtil, 2014; Johnson, 2017). Phospholipases catalyze the hydrolysis of phospholipids, which are common in mammalian membrane structures, to release fatty acids and a phosphoric acid mixture (Flores-Diaz et al., 2016; Hiller et al., 2018; Wilson and Knoll, 2018). Thus, Mtb phospholipases may play a crucial role in generating cell wall (CW) components and are necessary for Mtb adaptation and survival within macrophages, especially in blocking phagosomal maturation and killing (Santucci et al., 2018b; Sundar et al., 2019). The free fatty acids hydrolyzed by phospholipases provide an energy source for Mtb to grow and replicate in cells (Rameshwaram et al., 2018). However, current understanding of the gene(s), biological activity, and pathogenic mechanisms of phospholipases in Mtb are limited.

Patatins are glycosylated proteins that were first reported in potato tubers (Andrews et al., 1988; Shewry, 2003; Kienesberger et al., 2009). The protein sequence of PLP is similar to patatin and exhibits lipid acyl hydrolase activity (Banerji and Flieger, 2004; Kienesberger et al., 2009). The patatin-like catalytic domain, which has enzymatic activity that is dependent on a serine-aspartate dyad and an anion binding box, widely occurs among prokaryotes and eukaryotes. In many reports, PLP has been proposed to have phospholipase and lipase activity. PLA_1_ and PLA_2_, respectively, induce the accumulation of free fatty acids and 2-acyl lysophospholipids or 1-acyl lysophospholipids (Istivan and Coloe, 2006). Lipases catalyze the hydrolysis of ester bonds in long-chain acylglycerols to release fatty acids and glycerol. ScTgl3p, ScTgl4p, and ScTgl5p are PLPs that exhibit triacylglycerol (TAG) lipase activity in *Saccharomyces cerevisiae* to release TAG from lipid storage particles (Athenstaedt and Daum, 2003, 2005; Wagner and Daum, 2005). PLPs released by pathogenic microorganisms during the infection of a mammalian host have been implicated in nutrient acquisition, tissue invasion, and modulation of the host immune response (Kohler et al., 2006). RT0590 and RT0522, which are PLPs in *Rickettsia typhi*, exhibit PLA_2_ activity that requires a host cofactor and are cytotoxic to yeast cells. Furthermore, these also promote infection and phagocytic escape during the initial stages of infection (Rahman et al., 2010, 2013). *Legionella pneumophila* secretes a PLP, namely, VipD (with PLA_1_ activity), which robustly interferes with endosomal trafficking by selectively interacting with the Rab5‐ and Rab22-mediated removal of PI(3)P from endosomal membranes that consequently blocks endosomal trafficking, fusion with *Legionella*-containing vacuoles, and then lysosomal degradation of endocytic materials in macrophage cells (Ku et al., 2012; Gaspar and Machner, 2014). ExoU is a PLP virulence factor that exhibits PLA_2_ activity in the Gram-negative species *Pseudomonas aeruginosa*. The ubiquitination of this protein results in its co-localization with endosomal markers and rapid cell necrosis (Stirling et al., 2006; Diaz and Hauser, 2010; Gendrin et al., 2012). PlpD is a PLP in *P. aeruginosa* with both PLA_1_ activity and lipase activity, which is associated with an intracellular lifestyle.

The Mtb genome contains a PLP that has been described based on the occurrence of four conserved sequence blocks, which is a hallmark of the α/β hydrolase-fold family (Kuhle and Flieger, 2014). We study here the *rv3091* gene of Mtb H37Rv, which has been previously described as a putative patatin-like protein superfamily gene in National Center for Biotechnology Information (NCBI; Wijeyesakere et al., 2014; Madeira et al., 2016). It was analyzed *via* bioinformatics and comparative genomics in order to understand the important evolutionary characteristics of Rv3091. In addition, we also identified and characterized the phospholipase and lipase activity of the purified Rv3091 protein, which was not only overexpressed in *M. smegmatis* but also examined in terms of its role in its phagosomal resistance and intracellular survival to macrophages and C57BL/6 J mice. Collectively, these data indicate that Rv3091 is relevant to mycobacterial pathogenicity, and we also provide insights into the intracellular lifestyle of Mtb as mediated by Rv3091.

## Materials and Methods

### Cells and Animals

The human leukemic monocyte lymphoma cell line THP-1 and Vero (African green monkey kidney) cells were purchased from the ATCC (Manassas, VA, USA), which is maintained in our laboratory. Female specific pathogen-free (SPF) C57BL/6 J (6-week-old) mice were obtained from Changsheng Biotechnology Co., Ltd. (Liaoning, China) and housed in a pathogen-free, autoclaved micro-isolator cages at the Harbin Veterinary Research Institute of the Chinese Academy of Agricultural Sciences (Harbin, China). These mice were cared for and used, following the policies and regulations issued by the World Organization for Animal Health.

### Bacterial Strains and Culture Media

*Escherichia coli* DH5α (REF: 18265017, Invitrogen, USA) were grown on Luria-Bertani (LB) broth or agar (BD Biosciences, USA) supplemented with Amp^+^ (ampicillin, Sigma-Aldrich, USA) or Kan^+^ (kanamycin Sigma-Aldrich, USA) at a concentration of 50 μg/ml. *M. smegmatis* mc^2^155 (ATCC 700084) and *Mycobacterium bovis* BCG (ATCC 19274) were grown in Middlebrook 7H9 (BD Biosciences, USA) or Middlebrook 7H10 medium (BD Biosciences, USA) in the presence or absence of Kan^+^ at 50 μg/ml. Middlebrook 7H9 was used with 10% OADC (oleic acid/albumin/dextrose/catalase enrichment, BD Biosciences, USA), 0.2% glycerol (Sigma-Aldrich, USA), and 0.05% Tween 80 (Sigma-Aldrich, USA).

### Bioinformatics Analysis

Amino acid nucleic and acid sequences of Rv3091 in Mtb H37Rv were downloaded from NCBI^1^. The transmembrane helices of Rv3091 were predicted by TMHMM^2^. The signal peptide of Rv3091 was predicted by SignalP^3^. Conserved domains within Rv3091 were determined using NCBI Structure^4^. Amino acid composition, theoretical molecular weight, isoelectric point, classification of amino acid composition, positive, negative charge residues, instability coefficient, and theoretical half-life of Rv3091 were predicted and analyzed by ExPASy^5^. Sequence alignment and evolutionary analysis of proteins were performed using MEGA (version 7.0) with reference to the known bacterial PLPs, and conserved regions in the definition box were allocated to the alignment file by Espript 3.

### Molecular Modeling and Refinement

The three-dimensional (3-D) model of Rv3091 was built using SWISS-MODEL^6^ as described elsewhere (Cao et al., 2015). Briefly, the amino acid sequence of Rv3091 was uploaded to SWISS-MODEL. Built models were screened using PyMol^7^ to select the lowest root-mean-square deviation (RMSD) value. This had sequence identity with Rv3091 of the template used, *P. aeruginosa* PlpD (ARG85808.1, PDB 5fya.1; Madeira et al., 2016). The amino acid homology of Rv3091 and PlpD was 11.91%.

### Construction of *M. smegmatis* Recombinant Rv3091 Expression and Site-Directed Mutagenesis Strains

The *rv3091* gene was obtained from Mtb H37Rv (ATCC 25618) genome using primers 3091-261F and 3091-261R (Supplementary Table S1). The primers were designed according to the sequence (GenBank: CP003248.2) from NCBI. The *rv3091* gene was cloned into pMV261 (an *E. coli*-Mycobacteria shuttle vector created in our lab and vector sequence is shown in Supplementary Material) to obtain recombinant plasmid pMV261-Rv3091. The pMV261-Rv3091 plasmid and empty vector pMV261 were transformed into *M. smegmatis* mc^2^155. The positive transformants were confirmed by Kan^+^ resistance screening and western blot analysis use the anti-Rv3091 antibody (Rv3091 protein was expressed and purified from *E. coli* and inoculated into rabbits to obtain anti-Rv3091). The recombinant *M. smegmatis* (rMs) strains were designated as Ms3091 and Ms261. The pMV261-Rv3091 plasmid was amplified with mutagenic oligonucleotide primers (Supplementary Table S1) to introduce Ser-214-Ala, Asp-407-Ala, and both Ser-214-Ala Asp-407-Ala substitutions into the nucleotide sequence. High-fidelity polymerase (PrimeSTAR Max, TaKaRa Bio, Japan) was used for PCR amplification. The PCR products were digested with the restriction endonucleases *Dpn*I (Thermo Fisher Scientific, USA) to destroy the methylated DNA. The mutated plasmids were electroporated into *M. smegmatis* mc^2^155. The rMs was designated as Ms3091S214A, Ms3091D407A, and Ms3091S214AD407A. All of the site-directed mutageneses were verified by DNA sequencing.

### Analysis of the Subcellular Localization of Rv3091 Protein in Mycobacterium Cells

Subcellular fractionation of rMs and *M. bovis* BCG was conducted as described elsewhere with minor modifications (Vizcaino et al., 2010). Briefly, mycobacterium cells at logarithmic growth were harvested by centrifugation at 10,000 *g* for 30 min at 4°C. The supernatant was filtered through a 0.22-μm filter (Merck Millipore, USA) and ultrafiltered to generate the culture filtrate (CF) fraction. The 10-g (wet weight) pellet of mycobacteria was suspended in 20 ml of ice-cold 1 × PBS (pH 7.4) with nuclease and protease inhibitors (1 mM PMSF, 1 mM EDTA, 1 mg/ml leupeptin, 50 U/ml DNase, and 100 U/ml RNase, Roche, USA). The supernatant was centrifuged at 27,000 *g* for 60 min at 4°C. Then, the pellet was redissolved in 1 × PBS (pH 7.4) with 1 mM PMSF and 2 mM lysozyme (Sigma-Aldrich, USA). The suspension was incubated at 160 rpm for 60 min at 37°C and then centrifuged at 27,000 *g* for 1 h at 4°C. The pellet was considered as the CW fraction. The supernatant was pooled together with the fraction that was collected from the above-described centrifugation and was centrifuged at 100,000 *g* for 4 h at 4°C. This pellet was designated as the cell membrane (CM) fraction. The cytoplasmic fraction was the supernatant obtained from this centrifugation. The above isolated proteins were dissolved in 10 mM ammonium bicarbonate and quantified using the Micro BCA kit (Thermo Fisher Scientific, USA) and stored at −80°C. Western blotting of each fraction was performed using a rabbit polyclonal anti-Rv3091 (Rv3091 protein was expressed and purified from *E. coli* and inoculated into rabbits to obtain anti-Rv3091). As the Mtb KatG protein was only present in the CP, rabbit polyclonal anti-KatG (which was kept in our laboratory; Dang et al., 2018) was used to verify the accuracy of the component separation of this mycobacterium. The CF was concentrated 200 times and then subjected to mass spectrometry (Huada Technology Service Co., Ltd., Shenzhen, China).

### Expression and Purification of Rv3091 From rMs

The expression and purification of protein from rMs was performed as described elsewhere with minor modifications (Cao et al., 2015). The rMs (Ms3091, Ms3091S214A, Ms3091D407A, and Ms3091S214AD407A) strains were grown in 7H9 medium supplemented with 0.2% glycerol, 0.05% Tween 80, 10% OADC, and 50 μg/ml Kan^+^ at 37°C with a shaking speed of 160 rpm. Acetamide (Sigma-Aldrich) was added to the media at a final concentration of 0.4% (w/v) when the rMs cultures reached an OD_600_ of 0.8–1.5, and then the cultures were grown for another 24 h in order to allow for the expression of His-tag recombinant proteins. Cells were harvested by centrifugation at 5,000 rpm for 20 min at 4°C. The cells were resuspended in ice-cold washing buffer (10% glycerol, 20 mM Tris-HCl, 150 mM NaCl, pH 8.0) and disrupted by ultrasound at 4°C (Ultrasonic Crusher ULTRASONIC, Cole Parmer, USA). The supernatant was collected by centrifugation at 15,000 rpm for 30 min at 4°C and then loaded onto a Ni^2+^ Sepharose 6 Fast Flow column (GE Healthcare, USA) equilibrated with buffer A (20 mM Tris-HCl, 150 mM NaCl, 10 mM imidazole, pH 7.0) after being passed through a filter membrane (Millipore, 0.22 μm, USA). The column was washed with 10 column volumes of buffer A and an imidazole (Millipore, USA) concentration gradient (20, 40, 60, 80, and 100 mM). Proteins were eluted with buffer A containing 250 mM imidazole and 20% glycerol. The purified protein was then further purified using an anion exchange chromatography RESOURCETM Q column (1 ml; GE Healthcare, USA) and a Sephacryl™ S-100 HR column (GE Healthcare, USA). The collected protein samples were analyzed by 4–20% sodium dodecyl sulfate polyacrylamide gel electrophoresis (SDS-PAGE), and the concentration was determined using a BCA kit (Thermo Fisher Scientific, Inc., CA, USA). The recombinant proteins purified from Ms3091, Ms3091S214A, Ms3091D407A, and Ms3091S214AD407A were designated as Rv3091NS, S214A, D407A, and S214AD407A, respectively.

### Lipase Assay

Lipase activity was assayed by measuring the amount of p-nitrophenol (pNP) released from pNP ester substrates with varying lengths of fatty acids (Cao et al., 2015). The total lipase activity was assayed using the purified protein from recombinant *M. smegmatis*. The standard lipase activity assays were performed using a 100 μl reaction system (pH 8.0) consisting of a final concentration of 0.5 mM pNP esters [pNP-acetate (C2), pNP-butyrate (C4), pNP-caproate (C6), pNP-caprylate (C8), pNP-laurate (C12), pNP-myristate (C14), pNP-palmitate (C16), and pNP-stearate (C18)], protein (1, 2, 3, and 4 μg), 50 mM phosphate buffer, 80 mM H_3_BO_3_, 0.3% Triton X-100, and 20% glycerol. pNP esters were dissolved with isopropanol. The quantity of protein used in these experiments was 0.1 mg. Blank reaction mixture containing all components except for protein was also used. The reaction mix was incubated for 60 min at 37°C, and the reactions were then terminated by freezing for 15 min at −20°C. The release of pNP was estimated by measuring the absorbance at 405 nm using a BioTek ELx808 Absorbance Microplate Reader (BioTek, Winooski, United States). One unit of enzyme activity was defined as the amount of enzyme required to release 1 μmole of pNP per min at 37°C under standard reaction conditions. Enzyme activity (U/mg) = ΔA405 × K × V × 1,000/(T × ε). “A405” was the absorbance of the reaction solution. “K” was the dilution multiple of the enzyme; “V” was the volume of the reaction solution (ml); “T” was the reaction time (min); *ε* = 0.016 (μmol/L). The mutated proteins (S214AD407A 0.1 mg) and BSA (0.1 mg) were used as control groups for lipase activity determination. All reactions were conducted in triplicate (Chen et al., 2014; Cao et al., 2015; Kaur and Kaur, 2019).

### Phospholipase Assays

Phospholipase activity was assessed using the fresh yolk plate assay, as described elsewhere with minor modifications (Song et al., 1999). Briefly, the modified yolk agar plate contained 0.66% of NaCl, 0.1 M CaCl_2_, 1.09% of boric acid, 0.19% borax, 1.5% agar, 1.0% peptone, and 0.5% yeast extract. The plates were sterilized by autoclaving at 121°C for 15 min. Once the plates cooled to 55°C, fresh egg yolk was added to a final concentration of 15% (w/v) and then mixed thoroughly to completely emulsify the egg yolk. The mixture was used to prepare the reporter plates for assessment of phospholipase activity.

The PLA_1_ and PLA_2_ activity of purified recombinant protein (Rv3091NS, S214A, D407A, and S214AD407A) was detected using the fluorogenic phospholipid substrate [PLA_1_ and PLA_2_ substrates PED-A1 (Invitrogen, USA) and BODIPY PC-A2 (Invitrogen, USA)] according to the manufacturer’s protocol (EnzChek Phospholipase A_1_ Assay Kit and EnzChek Phospholipase A_2_ Assay Kit, Invitrogen, USA) as previously described with minor modifications (Trunk et al., 2019). Briefly, the cleavage of the *sn*-1/*sn*-2 bond of PED-A1/BODIPY PC-A_2_ using the PLA_1_/PLA_2_ enzyme induces an increase in fluorescence emission. Final concentration of the fluorescent phospholipid substrate was 1 mM. The phospholipid-substrate mix was prepared by mixing 25 μl of 10 mM dioleoylphosphatidylcholine (DOPC, Invitrogen, USA), dioleoylphosphatidylglycerol (DOPG, Invitrogen, USA), and 25 μl of 1 mM fluorogenic phospholipid substrate in 5 ml of PLA_1_/PLA_2_ assay buffer (50 mM Tris-HCl, 100 mM NaCl, 1 mM CaCl_2_, pH 7.4). Then, 50 μl of the substrate-liposome mix was added to 50 μl of PLA_1_/PLA_2_ samples. The assay buffer was 50 mM Tris-HCl, 100 mM NaCl, and 1 mM CaCl_2_, pH 7.4. The reaction mixtures were incubated at 25, 37, and 42°C for different durations. The fluorescence emission was then measured using an EnSpire Multiscan Spectrum (PerkinElmer, USA) at an excitation wavelength of 460 nm and an emission wavelength of 515 nm. The standard of phospholipase A_1_/A_2_ was component D of the EnzChek Phospholipase A_1_/A_2_ Assay Kit. A PLA_1_/PLA_2_ standard curve was prepared by diluting the appropriate amount of 500 Units/ml component D stock solution to 5 Unit/ml in the assay buffer (50 mM Tris-HCl, 100 mM NaCl, 1 mM CaCl_2_, pH 7.4) to produce PLA_1_/PLA_2_ concentrations of 0–5 Unit/ml. One unit of enzyme activity was defined as the amount of enzyme required to release 1 μmole of 4,4-difluoro-5,7-dimethyl-4-bora-3a,4a-diaza-s-indacene-3-pentanoic acid per min under standard reaction conditions. Specific activity was expressed in U/mg of protein. All reactions were conducted in triplicate.

For the PLA_1_ and PLA_2_ inhibitor assay, methyl arachidonyl fluorophosphonate (MAFP, Sigma-Aldrich) was used at a final concentration of 0.5 μM. For the PLA_1_ and PLA_2_ eukaryotic activator assay, Vero 76 cell lysate was added at a final concentration of 0.5 mg/ml. For the antibody blocking assay, anti-Rv3091 antibody or pre-immune IgG (unimmunized negative rabbit serum IgG) was incubated with Rv3091NS at a final concentration of 1, 2, and 5 mg/ml at 37°C for 30 min.

### Murine Peritoneal Macrophages Isolation

Murine peritoneal macrophages were collected as described elsewhere (Ray and Dittel, 2010). The C57BL/6 J (6-week-old) female mice were injected intraperitoneally with 2 ml of 3% (wt/vol) sterile sodium thioglycolate medium (MERCK, Darmstadt, Germany). Murine peritoneal macrophages were isolated after 3 days as follows: first, the mice were euthanized, followed by sterilization in 75% ethanol for 5 min, and then fixed on the dissection plates with the ventral side facing up. Next, the mice were dissected to expose the intact peritoneum, and 5 ml of ice cold RPMI 1640 (Gibco, Thermo Scientific, USA) were injected into the abdominal cavity. With gentle massaging, the cells in intraperitoneal RPMI 1640 medium were aspirated and collected by centrifugation at 1,000 rpm for 3 min. Finally, the collected cells were dispersed, counted, and cultured in RPMI 1640 with 10% FBS (Gibco, Thermo Scientific, USA).

### Intracellular Survival of rMs in Murine Peritoneal Macrophages

Freshly isolated murine peritoneal macrophages were cultured in 24-well cell culture plates (Corning, USA) at a density of 3 × 10^5^ cells/well in RPMI 1640 with 10% FBS and incubated at 37°C under 5% CO_2_. After 1 day, the cells were washed thrice with warm RPMI 1640 to remove any non-adherent cells. The adherent cells were cultured under the same conditions for another 2 days. rMs were grown until logarithmic phase in liquid 7H9 medium, and acetamide was added to the media for 1 day to allow for the expression of recombinant proteins, then each group of rMs was washed and resuspended three times. Cells were then infected with rMs on the third day. Regulation of bacteria at a multiplicity of infection (MOI) of 5 for macrophage infection. Following 2 h of incubation at 37°C in 5% CO_2_ atmosphere, the cells were washed thrice with warm RPMI 1640, and then, the extracellular bacteria were killed by incubating in RPMI 1640 medium supplemented with gentamicin (200 μg/ml; Sigma-Aldrich, USA) for 2 h at 37°C and 5% CO_2_ atmosphere for 6 h. Next, the infected macrophages were washed with RPMI 1640 thrice and cultivated in RPMI 1640 with 10% FBS containing gentamicin (20 μg/ml). Macrophages were lysed in 1 × PBS (pH 7.4) with 1% Triton X-100 (Sigma-Aldrich, USA) at 6, 12, 24, 36, and 48 h after infection. The lysates were then serially diluted and spread onto 7H10 plates for subsequent colony forming unit (CFU) counting of rMs that survived within macrophages.

### Transmission Electron Microscopy

Murine peritoneal macrophages were cultured in 6-well cell culture plates (Corning, USA) and then infected with Ms3091 or Ms261 at an MOI of 5. The cells were prepared and observed for transmission electron microscopy (TEM) at 24 h after infection as previously described (Li et al., 2019). Briefly, infected cells were washed with ice-cold 1 × PBS (pH 7.4) three times and then subsequently fixed in 3% glutaraldehyde (Sigma-Aldrich, USA) and 1% osmium tetroxide (Sigma-Aldrich, USA). Next, the cells were dehydrated in a concentration gradient of acetone (Sigma-Aldrich, USA; 50, 70, 90, and 100%) at 4°C. The dehydrated cells were then incubated and polymerized with Eponate 12™ Embedding Kit with DMP-30 (Catalog No.GP18010, Ted Pella, USA). After resin polymerization at 70°C for 2 days, the samples were cut into 100-nm-thick slices using an Ultramicrotome (LEICA EM UC6, Germany) and collected on carbon-coated copper grids (SPI-Chem, West Chester, USA). The grids were stained in distilled water containing 1% uranyl acetate (SPI-Chem, West Chester, USA) and then lead citrate (SPI-Chem, West Chester, USA) for 15 min, followed by thorough washing and drying. Next, the cells were observed with a TEM (Hitachi H-7650, Tokyo, Japan) and visualized using the iTEM software system. The visualization parameters were as follows: acceleration voltage, 80 kV; filament voltage, 22 V; filament time, 20 s; and emission current, 10 μA. First of all, low-power observation and then high-power observation were used. The target cells were placed in the center of the screen, and the focus, brightness, and contrast were adjusted to make the TEM images crisp and clear. Finally, the TEM images were captured with a CCD camera (VELETA, Part No. T014-10000-01, EMSIS GmbH, Germany).

### Translocation Assay by Immunofluorescence Labeling

The pMV361 vector (Gene Optimal, Shanghai, China) with enhanced green fluorescent protein (eGFP), designated as pMV361-eGFP (Gene Optimal, Shanghai, China, the corresponding sequence is shown in the Supplementary Data) was electroporated into Ms3091. The positive transformants were identified by fluorescence microscopy and western blot analysis. The recombinant rMs was designated as Ms3091-eGFP. Two milligrams of anti-Rv3091 or pre-immune IgG (unimmunized negative rabbit serum IgG) were incubated with Ms3091-eGFP at 37°C for 30 min before immunization. Regulation of bacteria at an MOI of 5 after treating with 50 ng/ml phorbol myristate acetate (PMA, Sigma-Aldrich, USA)-differentiated THP-1 cells infection at 37°C under 5% CO_2_. At 1 day post-infection, the infected cells were washed three times with 1 × PBS (pH 7.4) and then fixed at 25°C for 30 min with 4% paraformaldehyde (Sigma-Aldrich, USA). The fixed cells were thoroughly washed and then permeabilized at 25°C for 30 min with 0.1% Triton X-100. For fixation, cells were blocked in 1.0% BSA (Sigma-Aldrich, USA) at 37°C for 2 h to block the nonspecific protein-binding sites. After that, the cells were incubated with monoclonal mouse anti-human lysosomal-associated membrane protein 1 (LAMP-1; Abcam Inc., Cambridge, UK; at 1:100 dilution) as the primary antibody. The anti-rat-Alexa Fluor-594 (Thermo Fisher Scientific, USA) was used as the secondary antibody. Cell nuclei were stained using Hoechst 33342 (Thermo Fisher Scientific, USA). The cells were observed under a confocal microscope (ZEISS LSM800, Carl Zeiss Corp., Germany). Each treatment was repeated three times.

### The Survival and Pathogenicity of Ms3091 in Mice *in vivo*

Recombinant *M. smegmatis* (Ms3091 and Ms261) strains after induced expression were harvested and evenly dispersed in PBST (1 × PBS with 0.05% Tween 80). C57BL/6 J female mice (6-week-old) were intranasally infected with 1 × 10^7^ CFU of rMs/mouse. The lungs, kidneys, spleens, and livers of euthanized mice were harvested and homogenized in order to assess the survival of the rMs in the organs at days 1, 4, 7, 14, and 21 after infection. The organ homogenates were then diluted serially and spread onto 7H10 plates supplemented with 50 μg/ml Kan^+^. The lungs, kidneys, spleens, and livers of infected mice were embedded in paraffin (Solarbio, Beijing, China) for histopathological analysis and cut into 3–5-μm thick sections. Sections were stained with hematoxylin and eosin (H&E; Solarbio, Beijing, China) or Ziehl-Neelsen Carbolfuchsin (acid-fast bacillus) according to standard protocols.

### Statistical Analysis

All statistical analyses were conducted using GraphPad Prism ver. 8.3 software. All results were presented as the mean ± standard error of the means (SEMs). One-way and two-way ANOVA were employed to analyze statistical significance. Differences were considered statistically significant when *p* < 0.05 (^*^p < 0.05, ^**^p < 0.01, ^***^p < 0.001).

## Results

### Analysis of Conserved Domains and Evolutionary Trees of Rv3091 Protein

**Figure 1:**
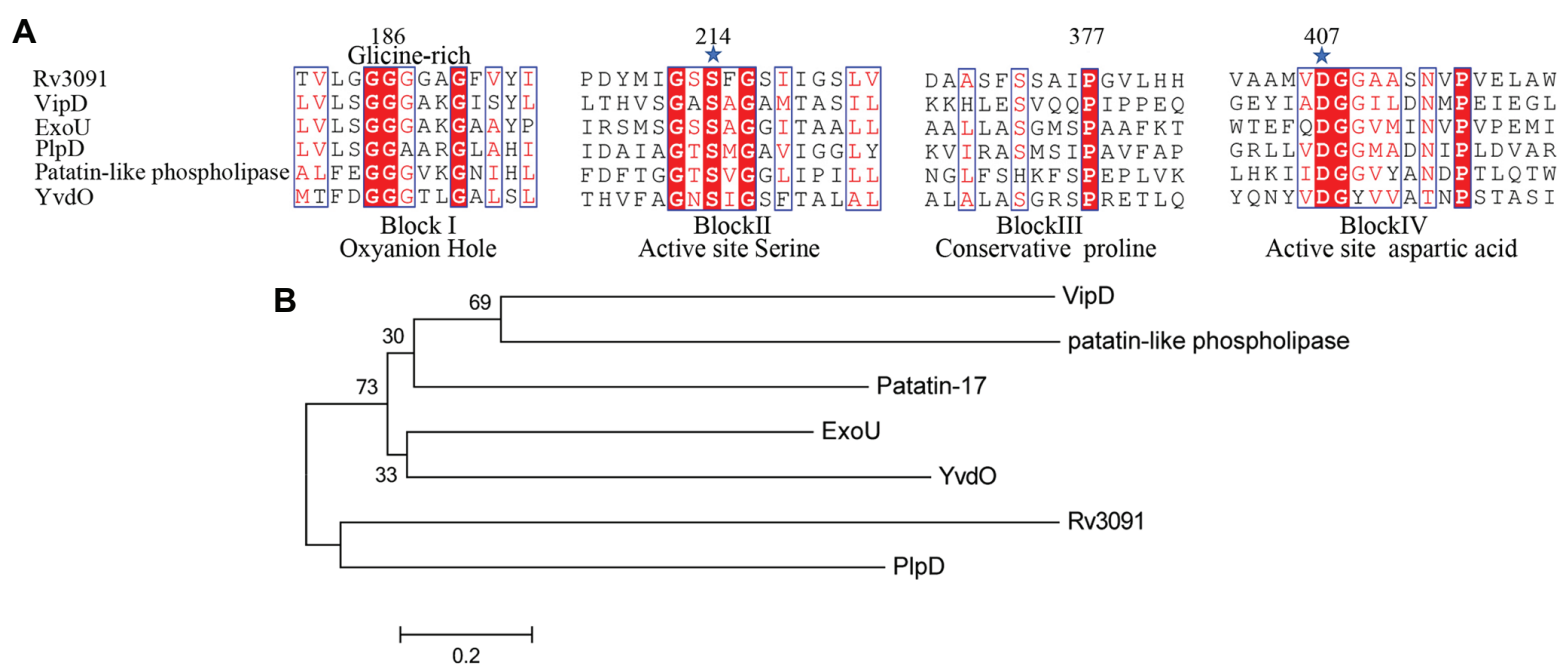
Biophysical informatics analysis of Rv3091 protein. **(A)** Multiple sequence alignments of the Rv3091 protein of *Mycobacterium tuberculosis* (Mtb) with other patatin-like phospholipases (PLPs): *Vibrio cholerae* Patatin-17 (TYC37870.1; Wijeyesakere et al., 2014), *Legionella pneumophila subsp*. VipD (YP_096826.1; Ku et al., 2012; Gaspar and Machner, 2014), *Pseudomonas aeruginosa* ExoU (ASM94169.1; Vanrheenen et al., 2006), *P. aeruginosa* PlpD (ARG85808.1; Madeira et al., 2016), *Rickettsia amblyommatis str.* PLP (AFC70055.1; Rahman et al., 2010), *Bacillus subtilis*. YvdO (QJR48018.1; Kato et al., 2010). The conserved catalytic residues Ser214 and Asp407 are indicated at the amino acid position by a star. **(B)** Phylogenetic tree of Rv3091 and other representative sequences of PLPs constructed using MEGA 7.0 software.

**Figure 2:**
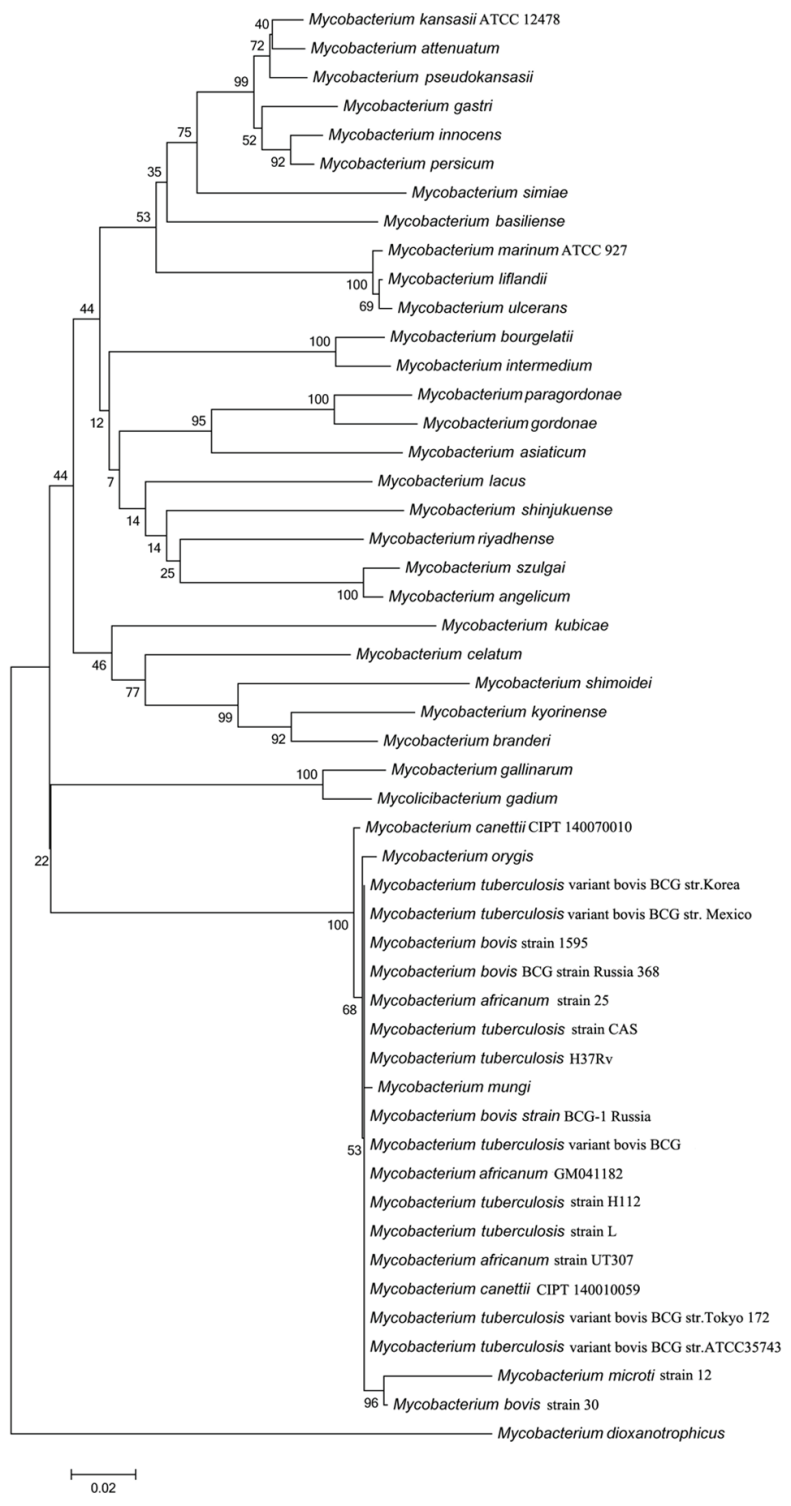
Evolutionary analysis of Rv3091 homologs proteins in mycobacteria using MEGA 7.0 software. Accession numbers for all sequences are provided in Supplementary Table S2.

The *rv3091* gene is 1,692 base pairs (bp) in length and encodes a protein of 564 amino acids. SignaIP 5.0 analysis did not identify a secretion signal sequence. The proteins that share consensus motifs are thought to be essential for patatin-like family of phospholipases (Vanrheenen et al., 2006; Wilson and Knoll, 2018), which include four conserved sequence blocks with four conserved residues, Gly186-Ser214-Gly377-Asp407, in blocks I, II, III, and IV (Figure 1A). The proteins of this family are known as PLPs (Madeira et al., 2016). Rv3091 consists of a stretch Gly186 proximal to the N-terminus within block I, which includes an oxyanion hole (Gly-Gly-X-Lys/Arg-Gly) that is a hallmark of PLPs. Block II consists of the active site Ser214 that is embedded within the well-known Gly-X-Ser-X-Gly motif. A conserved Ala-Ser-X-X-X-Pro motif is present in block III, and block IV contains an Asp407 active site embedded in an Asp407-Gly408 motif, which is unique among known bacterial lipases. Phylogenetic reconstruction of Mtb Rv3091 and other bacterial PLPs was performed using the maximum likelihood algorithm (Figure 1B). This model revealed that Mtb Rv3091 is closely related to PlpD of *P. aeruginosa* (Salacha et al., 2010; Chirani et al., 2018). Comparative analysis of mycobacteria genomes indicated that the Rv3091 homolog was a ubiquitous protein in mycobacteria that is likely essential for its biology (Gupta et al., 2018; Figure 2). They exist not only in pathogenic strains but also in non-pathogenic mycobacteria. However, Rv3091 homologs were shown to be absent from *Mycobacterium avium*. The chromosomal locations of *rv3091* are conserved across all mycobacteria genomes, suggesting that *rv3091* was present in the last common ancestor of the analyzed genomes. Rv3091 homolog proteins were conserved in sequence (81% average amino acid identity) with negligible length variations.

### 3-D Model Analysis of Rv3091 Protein

**Figure 3:**
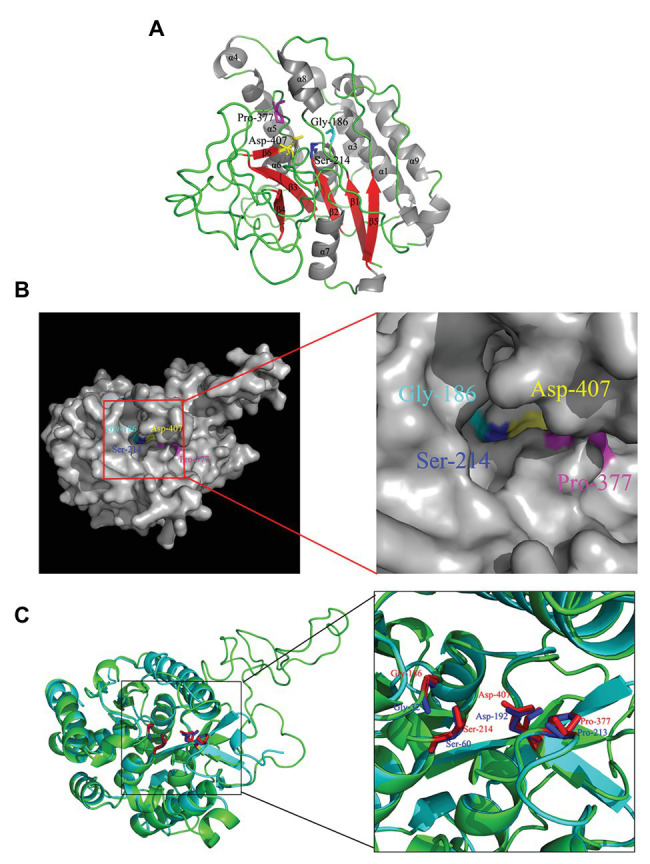
3-D model of Rv3091 protein. **(A)** 3-D structural modeling of Rv3091 reveals α/β hydrolase folds. The α helices are indicated in gray, β-strands in red, and loops in green. The conserved residue Gly186 is shown with cyan sticks, Ser214 is shown in blue sticks, Pro377 is shown in magenta sticks, and Asp407 is indicated as yellow sticks. **(B)** The surface representation of Rv3091. The conserved residue Gly186 is shown in cyan sticks, Ser214 is shown in blue sticks, Pro377 is shown in magenta sticks, and Asp407 is indicated in yellow sticks. It is noteworthy that the conserved residues formed a pocket surface structure. **(C)** Three-dimensional (3-D) structural alignment of Rv3091 and PlpD. The active site residues are shown in different colors in the 3-D structural model of Rv3091 and *P. aeruginosa* PlpD (red and blue, respectively).

Gly186, Ser214, Gly377, and Asp407 were identified to be strictly conserved residues of Rv3091 by biophysical characterization. To further obtain insights into the potential location of the catalytic residues in the overall topological organization of Rv3091, a 3-D model was constructed using SWISS MODEL. The final template we chose was PDB 5fya.1 (Madeira et al., 2016). As expected, the model revealed that Rv3091 consists of nine α helices and six β sheets (Figure 3A). Moreover, the center of the protein consisted of parallel β sheets, and the two sides of β sheets were covered with a number of α helices, forming a folding structure of alpha/beta/alpha. The surface representation of the conserved residues enabled us to better visualize the location of active site (Figure 3B). Conserved Gly186, Ser214, Gly377, and Asp407 residues in blocks I, II, III, and IV formed a hydrophobic pocket structure (Figure 3B; Madeira et al., 2016). Superimposition of the Rv3091 protein structure onto the PlpD crystal structure coincided with the predicted active site residues (Figure 3C). These results were consistent with Rv3091 being a patatin-like family phospholipase.

### Effect of Recombinant Rv3091 Overexpression in *M. smegmatis*

**Figure 4:**
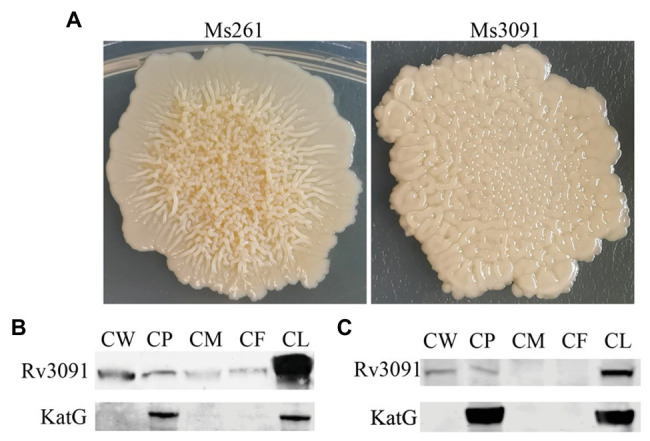
Subcellular localization of Rv3091 and its effects on colonymorphology of rMs. **(A)** Observation of single colony of Ms261 and Ms3091by magnification. **(B)** Subcellular localization of Rv3091 in Ms3091 using western blotting. **(C)** Subcellular localization of Rv3091 in *Mycobacterium bovis* BCG using western blotting. Culture filtrate (CF), whole-cell lysate products (CL), cell membrane (CM), cell wall (CW), cytoplasm (CP).

**Figure 5:**
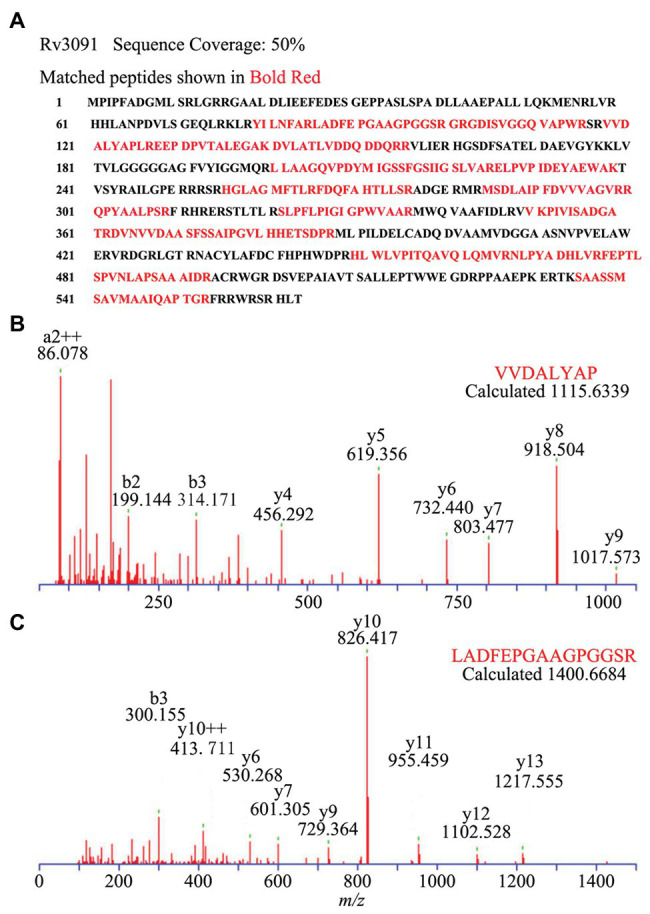
Subcellular localization of Rv3091 in culture filtrate of *M. bovis* BCG by Q-Excellent peptide mass fingerprint (PMF) spectrometry. **(A)** The sequence coverage of these fragments in Rv3091 is shown in bold red. **(B)** Doubly charged ion MS/MS of a fragmentation of Rv3091, VVDALYAP. **(C)** Doubly charged ion MS/MS of a fragmentation of Rv3091, LADFEPGAAGPGGSR.

We also constructed the overexpression strain of Rv3091 in *M. smegmatis* (Ms3091). We observed that the colony morphology of Ms3091 differed from that of the control Ms261 (Figure 4A). The Ms3091 was smooth with more regular, flat ridges, while recombinant Ms261 was rough with irregular ridges and folds. The cording phenotype of Ms3091 was less prominent than that of Ms261. To determine the reason for the morphological change of Ms3091, we investigated the subcellular location of Rv3091 within Ms3091. Subcellular fractions obtained from Ms3091 were hybridized with polyclonal antibodies specific to Rv3091. Our results showed that in Ms3091, Rv3091 protein bands were identified in all of the fractions (CW, CM, CP, and CF; Figure 4B). However, in *M. bovis* BCG, western blot analysis showed that Rv3091 was localized to the CW, CP (Figure 4C) and was further detected in CF by mass spectrometry (MS; Figure 5). The Q-Excellent peptide mass fingerprint spectrometry of peptides in *M. bovis* BCG CF was observed and submitted to Mascot. Mascot is software for identification, characterization, and quantitation of proteins using mass spectrometry data. Consequently, the Rv3091 protein from *M. tuberculosis* was obtained as a result with a score of 621. These results suggested that native Rv3091 is an extracellular protein of mycobacterium, and Ms3091 can be used as a model strain for studying Rv3091 protein function.

### Lipase Activity of Rv3091

**Figure 6:**
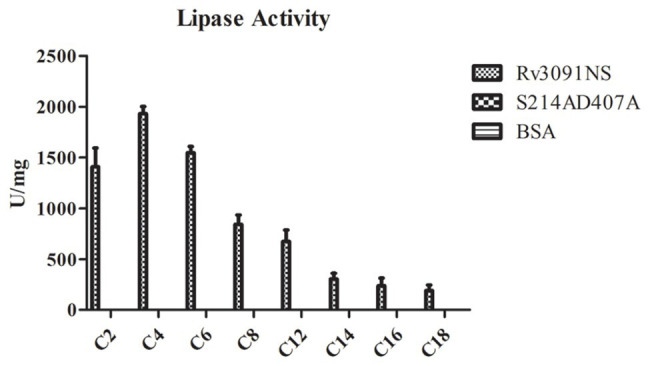
Lipase activity of Rv3091NS towards p-nitrophenol (pNP) with various chain lengths. C2: pNP-acetate, C4: pNP-butyrate, C8: pNP-caprylate, C10: pNP-capricate, C12: pNP-laurate, C14: pNP‐ myristate, C16: pNP-palmitate, C18: pNP‐ stearate. Mean ± SEMs (*n* = 3).

Some PLPs have been proposed to have lipase activity (Madeira et al., 2016). We investigated the potential lipase activity and substrate specificity of purified Rv3091 from rMs using a wide range of esters (pNPs), as substrates. The results showed that Rv3091 displayed high activity against both long-chain and short-chain pNP esters (Figure 6). The lipase activity of S214AD407A and BSA control groups were not detected (Figure 6). These data demonstrated that recombinant Rv3091 was a lipolytic enzyme of lipase in *M. smegmatis*.

### Phospholipase Activity of Recombinant Proteins

**Figure 7:**
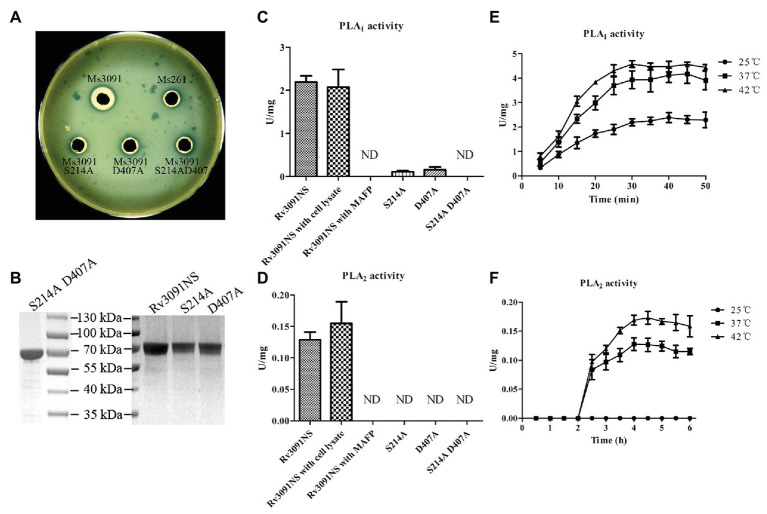
Evaluation of phospholipase activity of recombinant proteins. **(A)** The phospholipase activity of recombinant *M. smegmatis* (rMs) cell lysate supernatants on yolk agar plates. Ms3091, Ms3091S214A, Ms3091D407A, Ms3091S214AD407A, and Ms261 were spotted onto yolk agar plates for 24 h at 37°C. The creamy white zone in each halo represents the activity of phospholipase. **(B)** Sodium dodecyl sulfate polyacrylamide gel electrophoresis (SDS-PAGE) analysis of purified recombinant Rv3091NS, S214A, D407A, and S214AD407A proteins. **(C)** The PLA_1_ activities were measured at 25°C for 30 min. **(D)** The PLA_2_ activities were measured at 37°C for 4 h. **(E)** PLA_1_ activities were measured at 25, 37, and 42°C. **(F)** PLA_2_ activities were measured at 25, 37, and 42°C. ND, not detectable.

Both egg yolk broth and agar are most commonly used to screen bacteria for the production of phospholipases (Chrisope et al., 1976). Phospholipases can specifically hydrolyze phospholipids in egg yolk to produce free fatty acids. The fatty acids released are precipitated as calcium salts, forming the creamy white zones (Chrisope et al., 1976). Therefore, when the supernatant containing a phospholipase interacts with the egg yolk plate, a creamy white precipitation ring forms on the plate (Chrisope et al., 1976). Statistical analysis has shown that the diameter of the creamy white circle formed by the phospholipase activity is proportional to the activity of phospholipase. Based on this principle, our results showed that the diameter of the creamy white halo produced by Ms3091 cell lysate supernatants was 7 mm (Figure 7A). There was no obvious creamy white circle in the Ms3091S214A, Ms3091D407A, Ms3091S214AD407A, and control group (Ms261; Figure 7A). These results suggest that Rv3091 is a phospholipase.

To investigate the PLA activities of the Rv3091NS, S214A, D407A, and S214AD407A proteins, we harvested purified proteins from rMs (Ms3091, Ms3091S214A, Ms3091D407A, and Ms3091S214AD407A) using Ni^2+^ affinity chromatography (Figure 7B). The purified recombinant protein Rv3091NS displayed PLA_1_ and PLA_2_ activity (without Vero76 cell lysate; Figures 7C,D). Comparison of the site-directed mutagenesis purified proteins to wild-type Rv3091 demonstrated that the S214A substitution resulted in a ~95% loss of PLA_1_ activity and complete loss of PLA_2_ activity, the D407A substitutions resulted in a ~93% loss of PLA_1_ activity and complete loss of PLA_2_ activity, while the S214A and D407A double substitutions resulted in complete loss of PLA_1_ and PLA_2_ activity. MAFP (PLA_1_ and PLA_2_ inhibitor; Amara et al., 2012) inhibited the activity of Rv3091NS completely (Figures 7C,D). The PLA_1_ activity of Rv3091NS was enhanced at 42°C relative to tests conducted at 25 and 37°C (Figure 7E). The PLA_2_ activity of Rv3091NS was not detected at 25°C but increased significantly at 37 or even 42°C (Figure 7F). The above results showed that Rv3091 is a phospholipase protein with both PLA_1_ and PLA_2_ activity (Ser214 and Asp407 are active sites) at a relatively high temperature, which did not require the participation of cofactors for phospholipase activity.

### Rv3091 Enhanced the Survival of rMs in Macrophages

**Figure 8:**
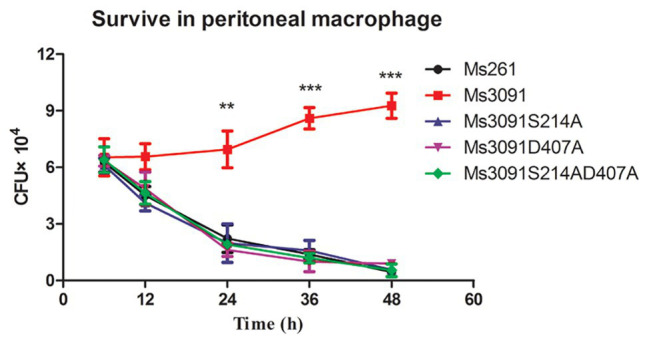
Intracellular survival of rMs (Ms261, Ms3091, Ms3091S214A, Ms3091D407A, and Ms3091S214AD407A) in murine peritoneal macrophages. Mean ± SEMs (*n* = 3), ^**^p < 0.01, ^***^p < 0.001.

To further study whether the PLA activity of Rv3091 was related to the invasion and intracellular viability of mycobacterium, we tested the impact of Rv3091 mutations on rMs and its ability to invade host cells *via* murine peritoneal macrophages. Our results indicate that Ms3091 significantly improves their survival in murine peritoneal macrophages (Figure 8). Bacterial intracellular survival was evaluated by counting the CFUs 6, 12, 24, 36, and 48 h after infection. Mutant and Ms261 exhibited a decrease in intracellular bacteria levels relative to Ms3091 12 h after infection (*p* > 0.05). Furthermore, cells infected with Ms3091 revealed 2.5-fold higher intracellular bacterial counts compared to those infected with the Ms3091S214A, Ms3091D407A, and Ms3091S214AD407A mutants at 24 h (*p* < 0.01). In addition, Ms3091 showed a significant increase in the number of CFUs relative to Ms261 at 24 h (*p* < 0.01), 36 h (*p* < 0.001), and 48 h (*p* < 0.001) post-infection. These findings show that Rv3091 plays an important role in the mycobacterium infectivity and survival at the cellular level.

### Rv3091 Promoted Mycobacterium Escape From Phagosomal Killing

There are many reasons for intracellular bacteria having the ability to escape phagosomes, of which one is believed to be PLA-mediated (Stirling et al., 2006; Rahman et al., 2013; Gaspar and Machner, 2014). PLA Rv3091 enhanced the intracellular survival of mycobacterium, but it was not known if it was related to phagosome escape. Accordingly, we examined the role Rv3091 plays in aiding the escape of mycobacterium from the phagosome into CP during the infection of rMs.

**Figure 9:**
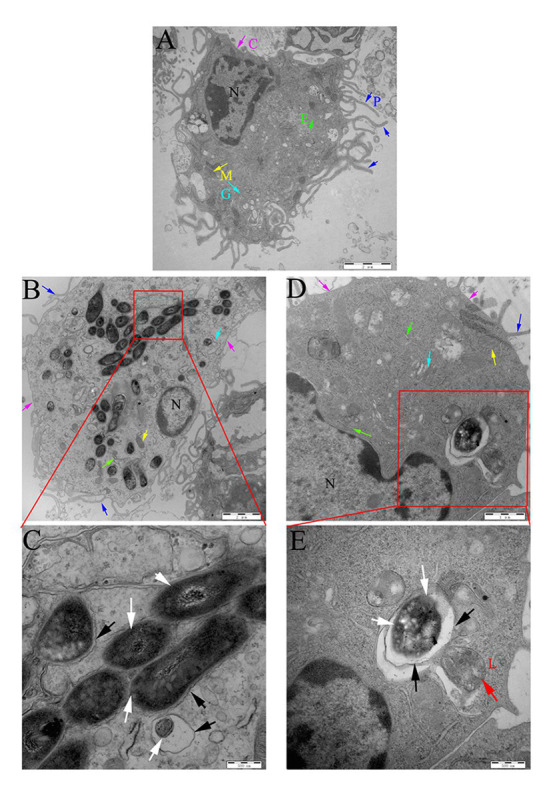
Localization of rMs (Ms261 and Ms3091) in murine peritoneal macrophages by transmission electron microscopy (TEM). **(A)** The ultrastructure of uninfected macrophages. Scale bar = 2 μm. **(B,C)** The ultrastructure of macrophages infected with Ms3091. The outlined areas in the B images (scale bar = 2 μm) are enlarged in the C images (scale bar = 500 nm). **(D,E)** The ultrastructure of macrophages infected with Ms261. The outlined areas in the D images (scale bar = 1 μm) are enlarged in E images (scale bar = 500 nm), lysosome (L, red arrow), nucleus (N), pseudopodia (P, blue arrows), cytoplasmic membrane (C, pink arrows), Golgi (G, cyan arrows), endoplasmic reticulum (E, green arrows), and mitochondrion (M, yellow arrows). Bacteria cell walls are marked with white arrows. Phagosomal membranes surrounding bacteria are marked with black arrows.

The peritoneal macrophages of mice were then infected with Ms3091 and Ms261. The intracellular localization of Ms3091 and Ms261 was assessed 24 h later by TEM. Uninfected macrophage controls were round or oval with abundant pseudopodia on the cell surface, and the CM had obvious integrity. The nucleus, endoplasmic reticulum, Golgi, mitochondria, and other organelles were normal in morphology, and the density of the CP was normal (Figure 9A). This was contrary to macrophages infected with the Ms261 control or Ms3091, which displayed a relatively intact nucleus, cytoplasmic membrane, and mitochondria (Figures 9B,D) but an endoplasmic reticulum and Golgi with obvious damage. In addition, the density of the CP was reduced in macrophages infected with Ms3091 (Figure 9B). Ms261 was mainly localized to membrane-bound vesicles, with only a small fraction being observed in the CP, and was easy to digest by combination with a lysosome (Figure 9E). However, most Ms3091 did not exhibit characteristic electron translucent zones or were not associated with any such vesicular structure (Figure 9C). This observation suggested that Ms3091 did not occur in membrane-enclosed compartments and instead was located in the cytosol. Together, these data suggested that Rv3091 promotes the escape of mycobacterium from the phagosome and increased rMs-dependent destruction of macrophages.

**Figure 10:**
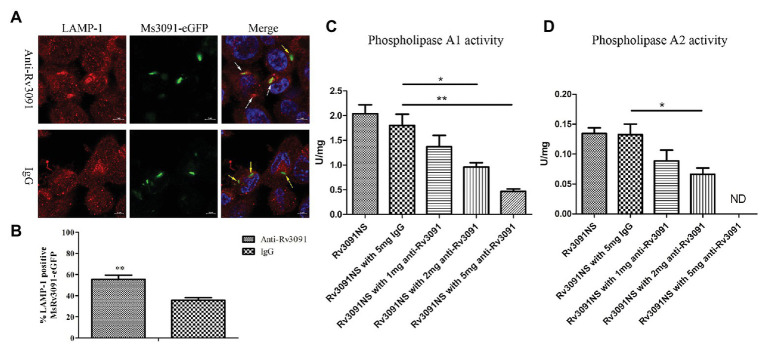
Rv3091 promotes rMs escape from phagosome killing in THP-1 cell. **(A)** Effect of anti-Rv3091 or pre-immune IgG pretreatment on Ms3091-eGFP phagosome escape by immunofluorescence. The white arrows indicate Ms3091-eGFP in LAMP-1-positive phagosomes, and Ms3091-eGFP in the process of escaping or escaped from the phagosome is indicated by yellow arrows. Scale bar = 5 μm. **(B)** Quantitation of the percentage Ms3091-eGFP in LAMP-1 positive phagosomes. **(C)** The PLA_1_ activity of Rv3091NS was measured after incubation with antibody at 25°C for 30 min. **(D)** The PLA_2_ activity of Rv3091NS was measured after incubation with antibody at 37°C for 4 h. ND, not detectable. IgG = pre-immune IgG, unimmunized negative rabbit serum IgG. More than 100 cells were assessed in three separate experiments. Mean ± SEMs, ^*^p < 0.05, ^**^p < 0.01.

PMA-differentiated THP-1 cells were infected by Ms3091-eGFP for 24 h. We monitored rMs phagosome escape using an immunofluorescence assay with an endosomal/lysosomal marker LAMP-1. Our findings indicated that Ms3091-eGFP pretreated with anti-Rv3091 antibodies mainly colocalized with the LAMP-1 marker (Figure 10A), indicating that Ms3091-eGFP is enclosed in the phagosome after 24 h of infection. However, Ms3091-eGFP pretreated with pre-immune IgG showed a relatively lower number of LAMP-1 positive signals at 24 h post-infection (Figure 10A), supporting Ms3091-eGFP escape from phagosome. Statistical analysis of counts showed that the percentage of Ms3091-eGFP hybridized to anti-Rv3091 antibody in LAMP-1 significantly increased compared to Ms3091-eGFP that was pretreated with pre-immune IgG (*p* < 0.01; Figure 10B). The activity of Rv3091NS after incubation with the anti-Rv3091 antibody was significantly reduced, and it was inversely proportional to the anti-Rv3091 antibody concentration (Figures 10C,D). The activity of Rv3091NS after incubation with pre-immune IgG was not significantly different from the activity of Rv3091NS unincubated with antibody (Figures 10C,D). Taken together, these findings showed that these antibodies blocked the activity of Rv3091 before host-macrophage infection, preventing rMs escape from phagosomes.

### The Role of Rv3091 in rMs Persistence in Lungs and Histopathological Analysis

**Figure 11:**
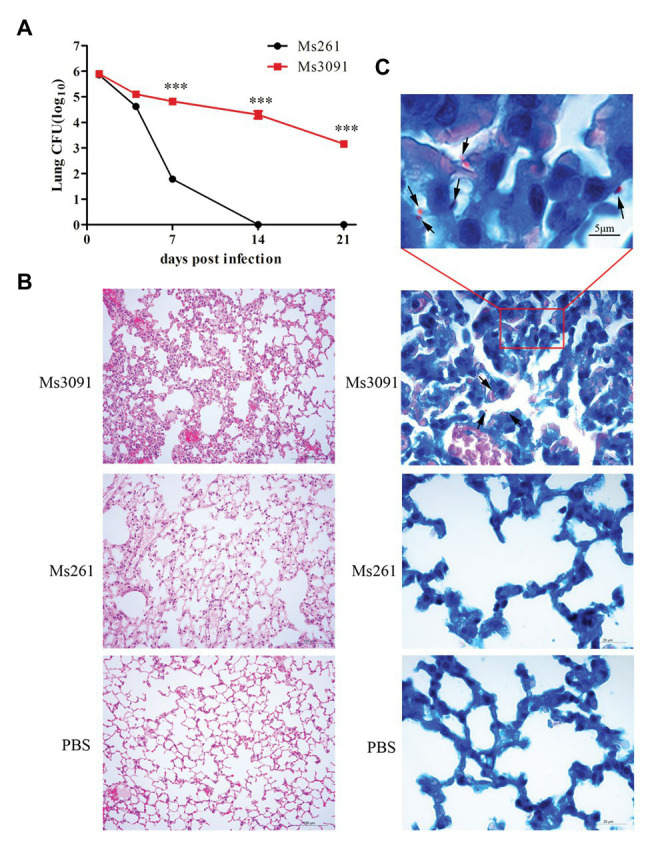
Bacterial loads, histopathological and acid-fast staining analysis of mouse lung. **(A)** Colony forming units (CFUs) in the lungs of C57BL/6 J mice. Means ± SEMs (*n* = 5), ^***^p < 0.001. **(B)** Histopathological analysis of mice lungs. C57BL/6 J mice were infected by intranasal infection with Ms261 and Ms3091 at 14 days, using 1 × PBS (pH 7.4) as a negative control. Scale bar = 100 μm. **(C)** Acid-fast staining analysis of mouse lung. C57BL/6 J mice were infected by intranasal infection with Ms261 and Ms3091 at 14 days, using 1 × PBS (pH 7.4) as a negative control. The outlined areas in the bottom images (scale bar = 20 μm) are enlarged in the top images (scale bar = 5 μm). Ms3091 are marked with black arrows.

We next performed *in vivo* infection experiments to further understand the role of Rv3091 in persistence. For the control Ms261, the abundance of rMs in the lungs of C57BL/6 J mice rapidly decreased as early as the first week and even completely cleared 2 weeks after infection (Figure 11A). However, the rMs burden in the lungs of the Ms3091 group gradually decreased, and we continued to detect Ms3091 3 weeks post infection (Figure 11A). Furthermore, the rMs burden in the Ms3091 lungs was significantly higher relative to the control Ms261 group 1–3 weeks after infection (*p* < 0.001; Figure 11A). Furthermore, we did not detect any rMs in the intestine, liver, spleen, or kidneys of C57BL/6 J mice. Only the lungs were inflamed, and the spleen, intestine, liver, and kidneys had no macroscopic lesions. The histological results showed that the Ms261 group had no pathological changes and no red rod-shaped bacteria after acid-fast staining in the lungs (Figures 11B,C). However, partial mild extravasation of blood and hyperplasia was observed in the alveolar epithelial cells in the Ms3091 group (Figure 11B). In addition, it can be seen that red rod-shaped acid-fast staining positive bacteria were located in the alveolar cavity and the CP of alveolar epithelial cells (Figure 11C). Therefore, our histopathological and acid-fast staining observations of infected mice coincided with the findings of bacterial burden in mouse organs. These results suggest that the PLA activity of Rv3091 was essential to rMs to resist defensive clearance in the lungs and was related to mycobacterium pathogenicity.

## Discussion

A few previous reports, which described the PLA in Mtb, mainly focused on activity and structural research, and there are no data currently available regarding PLP activity and pathogenicity (Parker et al., 2007, 2009; Sundar et al., 2019). In our investigation, we have, for the first time, successfully constructed a Rv3091 recombinant *M. smegmatis* (Ms3091) and identified that the extracellular activity of Rv3091 promoted escape from the phagosome and intracellular survival of *M. smegmatis*. These results indicate that the PLP Rv3091 confers pathogenicity to *M. smegmatis* and may act as a virulence factor of Mtb.

The amino acid sequence of Rv3091 shows significant sequence homology to the PLP domain of *P. aeruginosa* PlpD (PLA_1_ and lipase activity), *P. aeruginosa* ExoU (PLA_2_ activity), and *L. pneumophila* VipD (PLA_1_ and/or PLA_2_ activity), a virulence factor with PLA activity (Vanrheenen et al., 2006; Salacha et al., 2010; Ku et al., 2012). To identify which motif is responsible for the PLP activity in Rv3091, site-directed mutagenesis was conducted. Detection of the PLA activity in the mutants revealed that the S214A, D407A, and S214AD407A recombinant proteins showed a significant decrease in the PLA_1_ and PLA_2_ activity relative to the wild-type protein. Through homologous modeling, we found that the catalytic residues Ser214 and Asp407 in blocks II and IV formed the serine-aspartate dyad structure. An anion binding box within block I of the catalytic residues is designated as an oxyanion hole motif, which is concordant with the findings of an earlier study (Salacha et al., 2010; Madeira et al., 2016). Block IV comprises the second amino acid of a catalytic dyad, namely, an aspartate that is embedded in an aspartate-glycine motif, which exclusively occurs among known bacterial lipases (Kuhle and Flieger, 2014). We also report the overall topological organization of Rv3091 using homologous modeling, and the putative active center agrees with the experimental results of our site-directed mutagenesis assay. This finding improves information on important reference values for the in-depth analysis of Rv3091 structure and function.

The overexpression of the *rv3091* gene induces a significant alteration in the morphology phenotype in *M. smegmatis*. Similar to earlier studies, the overexpression of genes, including *rv1818c*, *rv1169c*, *rv0774c*, and *rv0518*, changes the colony morphology of *M. smegmatis* (Delogu et al., 2004; Singh et al., 2016; Kumar et al., 2017; Kaur and Kaur, 2019). This alteration may be attributable to any changes to the lipolytic activity involving CW lipids (Singh et al., 2016), suggesting that the PLP Rv3091 is involved in CW lipid remodeling that results in altered morphology (Kumar et al., 2017). Colony morphology has been associated with *mycobacterium* virulence, intracellular survival, immune modulation, drug resistance, infection, and altered signaling pathways (Kuze and Uchihira, 1984; Shiratsuchi et al., 1993; Tse et al., 2002). Therefore, the change in colony morphology of Ms3091 suggests that Rv3091 may be a key protein involved in the survival and pathogenicity of mycobacterium.

Previous studies have shown that the enzymatic activity of many PLPs is dependent upon a eukaryotic cofactor. Similarly, PLA_2_ of ExoU, RP534, RT0522, and RT0590 is dependent on superoxide dismutase (SOD1; Anderson et al., 2011; Housley et al., 2011; Rahman et al., 2013). PLA_1_ of VipD is dependent on Rab5 and Rab22 (Ku et al., 2012). In this study, activity characterization revealed that unlike many other PLPs, Rv3091 exhibited PLA_1_ activity in addition to PLA_2_ activity and did not need the participation of eukaryotic cofactors, which is consistent with the PLP AhPIA and BpPIA from the intracellular pathogens *Aeromonas hydrophila* and *Burkholderia pseudomallei* that display both PLA_1_ and PLA_2_ activity (Trunk et al., 2019). We failed to detect robust PLA_2_ activity in Rv3091 as compared with PLA_1_ activity. This phenomenon may be attributed to the fact that low PLA_2_ activity is essential to support the bacterial obligate intracellular growth in host cells without inducing any rapid damage (at least until bacteria induce host cell lysis to promote dispersal). Additionally, these are associated with beneficial functions to bacteria but do not substantially impact host fitness, which is concordant with the findings of previous studies (Rahman et al., 2010). Activity characterization revealed that the PLA_1_ and PLA_2_ activity of Rv3091 was accentuated at 42°C when compared to tests performed at 25 and 37°C. This temperature range is concordant with human and animal body temperature during Mtb infection, suggesting that Rv3091 may be associated with mycobacterium virulence.

Patatin-like phospholipases have been implicated in infection of host cells and phagosome escape of various pathogenic bacteria (Rahman et al., 2010; Ku et al., 2012; Gaspar and Machner, 2014). However, the pathogenicity of this enzymatic activity in relation to known mycobacterium proteomes has not been examined to date. This study demonstrated by colony counting that Ms3091 significantly increased rMs infectivity in murine peritoneal macrophages cells. Furthermore, TEM analysis showed that Ms3091 was able to gain access to the CP as compared with control Ms261, where they multiplied. Pretreatment of Ms3091 with anti-Rv3091 antibodies negatively affected the escape of rMs from the phagosome by immunofluorescence. The reason for these results may be that Rv3091 uses PLA to hydrolyze and/or modify phospholipid(s) related to the killing function of phagocytes, which consequently inhibits the ability of phagocytes to kill. These observations suggested that Rv3091 was a key protein for macrophage survival and that it might play a vital role in promoting phagosome escape of mycobacterium, as previously reported for the *R. typhi* PLA_2_, Pat1, and Pat2 (Rahman et al., 2013). However, the survival of Mtb in host cells may also be due to their ability to metabolize stored lipids to obtain nutrients (Kovacic et al., 2013; Kaur et al., 2018). Rv3091 protein overexpression allow recombinant Ms3091 to use lipids as a major carbon source, which further supports the role of this protein in bacterial intracellular survival, which agrees with the findings of previous studies (Zambrano and Kolter, 2005; Wang et al., 2008; Santucci et al., 2018a). These results were consistent with previous studies (Kumar et al., 2017; Maan et al., 2018; Kaur and Kaur, 2019).

Our *in vivo* infection experiments revealed that Ms3091 persists in the lungs of mice after 21 days of infection. Analysis of bacterial burden in mice revealed that Rv3091 protein expression significantly prolonged rMs survival in the lungs of infected mice. Moreover, severe pathological lesions in the lungs of the Ms3091-infected group were correlated with elevated tissue bacterial burden. These findings reveal that Rv3091 serves as a virulence factor in mycobacterium and could be related to the persistence of mycobacterium within the host.

Taken together, we identify a novel virulence factor, Rv3091, in mycobacterium, which is an extracellular PLA belonging to the patatin-like family. Rv3091 exhibits the characteristic α/β fold of hydrolases, which is also confirmed by biochemical and biophysical assays. Rv3091 overexpression in the surrogate *M. smegmatis* enhances its survival capability and allows the escape of *M. smegmatis* from the phagosomes of macrophages. The involvement of the Rv3091 protein in mycobacterial pathogenicity was confirmed by *in vivo* infection in mice. The rMs strain that overexpressed the *rv3091* gene significantly enhanced bacterial burden and injury to the lungs of infected mice. These results suggested that the PLA activity of Rv3091 protein confers phagosomal resistance and enhances intracellular survival of macrophages to mycobacteria. Furthermore, this protein helped the bacteria utilize alternative lipids as a carbon source. Thus, our findings not only provide insights into the pathogenesis of mycobacterium but also highlights the Rv3091 protein as a novel target for the development of novel treatment schemes for TB.

## Data Availability Statement

All datasets presented in this study are included in the article/Supplementary Material.

## Ethics Statement

The animal study was reviewed and approved by Ethical permission was obtained from the Institutional Animal Care and Ethics Committee (IACEC) of Harbin Veterinary Research Institute (HVRI), Chinese Academy of Agricultural Sciences (CAAS; permit No. SY-2018-Mi-063).

## Author Contributions

ZC, GD, and SL contributed with conception of the project and experiment design. ZC performed experiments and generated data. NS, YC, and HL assisted in enzyme activity and TEM experiments. ZL, XZ, and ZW assisted in animal experiments. ZC, NS, and GD wrote the manuscript. All authors contributed to manuscript revision and approved the submitted version.

### Conflict of Interest

The authors declare that the research was conducted in the absence of any commercial or financial relationships that could be construed as a potential conflict of interest.

## Footnotes

^1^https://www.ncbi.nlm.nih.gov/

^2^http://www.cbs.dtu.dk/services/TMHMM/

^3^http://www.cbs.dtu.dk/services/SignalP/

^4^https://www.ncbi.nlm.nih.gov/Structure/cdd/wrpsb.cgi

^5^http://www.expasy.org/

^6^http://swissmodel.expasy.org/

^7^http://www.pymol.org/
