# Supplementary material for: Rv3091, An Extracellular Patatin-Like Phospholipase in Mycobacterium tuberculosis, Prolongs Intracellular Survival of Recombinant Mycolicibacterium smegmatis by Mediating Phagosomal Escape
Source: Front Microbiol. 2020 Sep 11;11:2204. doi: 10.3389/fmicb.2020.532371 (PMC7517356; doi:10.3389/fmicb.2020.532371)

***Supplementary Material***

**1. Supplementary Tables :**

Supplementary Table S1. Primers used for PCR of *rv3091* genes and its complementary mutagenic oligonucleotides used in this study.

| Primer | sequence 5’ 3’ |
| --- | --- |
| Rv3091-261F | CGC**GAATTC**ATGCCGATCCCCTTTGCCGA |
| Rv3091-261R | ATA**CATATG**TCAGGTGGCGGCTTCGCCATC |
| S214A-F: | CCCGACTACATGATCGGCTCGGCGTTCGGG |
| S214A-R: | CCGAGCCGCAAGCCCAGCTAGTAGCCGTCG |
| D407A-F: | CAGGACGTCGCGGCGATGGTCGCCGGCGGC |
| D407A-R: | TACCAGCGGCCGCCGCGCCGGTCGTTGCAG |
| The restriction sites are indicated in bold. | |

Supplementary Table S2. NCBI accession numbers for mycobacteria Rv3091 homologs sequences

| Rv3091 homologs sequences Taxon | NCBI |
| --- | --- |
| *Mycobacterium tuberculosis* H37Rv | NP_217607.1 |
| *Mycobacterium* *tuberculosis* strain L | QEX90927.1 |
| *Mycobacterium* *tuberculosis* strain H112 | AWY84259.1 |
| *Mycobacterium* *tuberculosis* strain CAS | AVV89124.1 |
| *Mycobacterium* *microti* strain 12 | AMC60832.1 |
| *Mycobacterium* *africanum* strain UT307 | AMQ39789.1 |
| *Mycobacterium* *africanum* GM041182 | CCC28168.1 |
| *Mycobacterium* *africanum* strain 25 | AMC65504.1 |
| *Mycobacterium* *canettii* CIPT 140010059 | CCC45448.1 |
| *Mycobacterium* *canettii* CIPT 140070010 | CCK61198.1 |
| *Mycobacterium* *mungi* | WP_064319938.1 |
| *Mycobacterium* *kansasii* ATCC 12478 | AGZ52789.1 |
| *Mycobacterium* *lacus* | WP_085160622.1 |
| *Mycobacterium* *gastri* | WP_036416962.1 |
| *Mycobacterium* *celatum* | WP_085168100.1 |
| *Mycobacterium* *innocens* | WP_075544140.1 |
| *Mycobacterium* *persicum* | WP_099223114.1 |
| *Mycobacterium* *orygis* | WP_003416089.1 |
| *Mycobacterium* *tuberculosis* variant bovis BCG | CUI13087.1 |
| *Mycobacterium* *bovis* BCG strain Russia 368 | ALA79675.1 |
| *Mycobacterium* *tuberculosis* variant bovis BCG str. Tokyo 172 | AMO11436.1 |
| *Mycobacterium* *bovis* strain BCG-1 (Russia) | ALV12032.1 |
| *Mycobacterium* *bovis* strain 1595 | AKR02964.1 |
| *Mycobacterium* *tuberculosis* variant bovis BCG str. ATCC 35743 | AHM08861.1 |
| *Mycobacterium* *tuberculosis* variant bovis BCG str. Mexico | AET20384.1 |
| *Mycobacterium* *tuberculosis* variant bovis BCG str. Korea 1168P | AGE69118.1 |
| Mycobacterium *bovis* strain 30 | AMC56535.1 |
| *Mycobacterium* *marinum* ATCC 927 | BBC64693.1 |
| *Mycobacterium* *kyorinense* | WP_045377525.1 |
| *Mycobacterium* *branderi* | WP_083130228.1 |
| Mycobacterium attenuatum | WP_122525207.1 |
| *Mycobacterium pseudokansasii* | WP_099188145.1 |
| *Mycobacterium bourgelatii* | WP_163709572.1 |
| *Mycobacterium simiae* | WP_149655673.1 |
| *Mycobacterium kubicae* | WP_068164087.1 |
| *Mycobacterium intermedium* | WP_079219849.1 |
| *Mycobacterium liflandii* | WP_015355015.1 |
| *Mycobacterium ulcerans* | WP_096370241.1 |
| *Mycobacterium shinjukuense* | WP_083046231.1 |
| *Mycobacterium szulgai* | WP_085669645.1 |
| *Mycobacterium angelicum* | WP_083112108.1 |
| *Mycobacterium paragordonae* | WP_133448780.1 |
| *Mycobacterium riyadhense* | WP_085249273.1 |
| *Mycobacterium asiaticum* | WP_036353971.1 |
| *Mycobacterium gallinarum* | WP_163725254.1 |
| *Mycobacterium shimoidei* | WP_069398247.1 |
| *Mycobacterium gordonae* | WP_069433445.1 |
| *Mycobacterium dioxanotrophicus* | WP_087082608.1 |
| *Mycobacterium basiliense* | WP_158015906.1 |
| *Mycolicibacterium gadium* | WP_163686402.1 |

1. **pMV261 vector sequence and multiple cloning site (MCS)**

AAAGCCACGTTGTGTCTCAAAATCTCTGATGTTACATTGCACAAGATAAAAATATATCATCATGAACAATAAAACTGTCTGCTTACATAAACAGTAATACAAGGGGTGTTATGAGCCATATTCAACGGGAAACGTCTTGCTCGAGGCCGCGATTAAATTCCAACATGGATGCTGATTTATATGGGTATAAATGGGCTCGCGATAATGTCGGGCAATCAGGTGCGACAATCTATCGCTTGTATGGGAAGCCCCATGCGCCAGAGTTGTTTCTGAAACATGGCAAAGGTAGCGTTGCCAATGATGTTACAGATGAGATGGTCAGACTAAACTGGCTGACGGAATTTATGCCTCTTCCGACCATCAAGCATTTTATCCGTACTCCTGATGATGCATGGTTACTCACCACTGCGATCCCCGGGAAAACAGCATTCCAGGTATTAGAAGAATATCCTGATTCAGGTGAAAATATTGTTGATGCGCTGGCAGTGTTCCTGCGCCGGTTGCATTCGATTCCTGTTTGTAATTGTCCTTTTAACAGCGATCGCGTATTTCGTCTCGCTCAGGCGCAATCACGAATGAATAACGGTTTGGTTGATGCGAGTGATTTTGATGACGAGCGTAATGGCTGGCCTGTTGAACAAGTCTGGAAAGAAATGCATAATCTTTTGCCATTCTCACCGGATTCAGTCGTCACTCATGGTGATTTCTCACTTGATAACCTTATTTTTGACGAGGGGAAATTAATAGGTTGTATTGATGTTGGACGAGTCGGAATCGCAGACCGATACCAGGATCTTGCCATCCTATGGAACTGCCTCGGTGAGTTTTCTCCTTCATTACAGAAACGGCTTTTTCAAAAATATGGTATTGATAATCCTGATATGAATAAATTGCAGTTTCATTTGATGCTCGATGAGTTTTTCTAATCAGAATTGGTTAATTGGTTGTAACACTGGCAGAGCATTACGCTGACTTGACGGGACGGCGGCTTTGTTGAATAAATCGAACTTTTGCTGAGTTGAAGGATCAGATCACGCATCTTCCCGACAACGCAGACCGTTCCGTGGCAAAGCAAAAGTTCAAAATCACCAACTGGTCCACCTACAACAAAGCTCTCATCAACCGTGGCTCCCTCACTTTCTGGCTGGATGATGGGGCGATTCAGGCCTGGTATGAGTCAGCAACACCTTCTTCACGAGGCAGACCTCACTAGTTCCACTGAGCGTCAGACCCCGTAGAAAAGATCAAAGGATCTTCTTGAGATCCTTTTTTTCTGCGCGTAATCTGCTGCTTGCAAACAAAAAAACCACCGCTACCAGCGGTGGTTTGTTTGCCGGATCAAGAGCTACCAACTCTTTTTCCGAAGGTAACTGGCTTCAGCAGAGCGCAGATACCAAATACTGTCCTTCTAGTGTAGCCGTAGTTAGGCCACCACTTCAAGAACTCTGTAGCACCGCCTACATACCTCGCTCTGCTAATCCTGTTACCAGTGGCTGCTGCCAGTGGCGATAAGTCGTGTCTTACCGGGTTGGACTCAAGACGATAGTTACCGGATAAGGCGCAGCGGTCGGGCTGAACGGGGGGTTCGTGCACACAGCCCAGCTTGGAGCGAACGACCTACACCGAACTGAGATACCTACAGCGTGAGCATTGAGAAAGCGCCACGCTTCCCGAAGGGAGAAAGGCGGACAGGTATCCGGTAAGCGGCAGGGTCGGAACAGGAGAGCGCACGAGGGAGCTTCCAGGGGGAAACGCCTGGTATCTTTATAGTCCTGTCGGGTTTCGCCACCTCTGACTTGAGCGTCGATTTTTGTGATGCTCGTCAGGGGGGCGGAGCCTATGGAAAAACGCCAGCAACGCGGCCTTTTTACGGTTCCTGGCCTTTTGCTGGCCTTTTGCTCACATGTTCTTTCCTGCGTTATCCCCTGATTCTGTGGATAACCGTATTACCGCCTTTGAGTGAGCTGATACCGCTCGCCGCAGCCGAACGACCGAGCGCAACGCGTGAGCCCACCAGCTCCGTAAGTTCGGGTGCTGTGTGGCTCGTACCCGCGCATTCAGGCGGCAGGGGGTCTAACGGGTCTAAGGCGGCGTGTACGGCCGCCACAGCGGCTCTTAGCGGCCCGGAAACGTCCTCGAAACGACGCATGTGTTCCTCCTGGTTGGTACAGGTGGTTGGGGGTGCTCGGCTGTCGCTGGTGTTTCATCATCAGGGCTCGACGGGAGAGCGGGGGAGTGTGCAGTTGTGGGGTGGCCCCTCAGCGAAATATCTGACTTGGAGCTCGTGTCGGACCATACACCGGTGATTAATCGTGGTTTATTATCAAGCGTGAGCCACGTCGCCGACGAATTTGAGCAGCTCTGGCTGCCGTACTGGTCCCTGGCAAGCGACGATCTGCTCGAGGGGATCTACCGCCAAAGCCGCGCGTCGGCCCTAGGCCGCCGGTACATCGAGGCGAACCCAACAGCGCTGGCAAACCTGCTGGTCGTGGACGTAGACCATCCAGACGCAGCGCTCCGAGCGCTCAGCGCCCGGGGGTCCCATCCGCTGCCCAACGCGATCGTGGGCAATCGCGCCAACGGCCACGCACACGCAGTGTGGGCACTCAACGCCCCTGTTCCACGCACCGAATACGCGCGGCGTAAGCCGCTCGCATACATGGCGGCGTGCGCCGAAGGCCTTCGGCGCGCCGTCGATGGCGACCGCAGTTACTCAGGCCTCATGACCAAAAACCCCGGCCACATCGCCTGGGAAACGGAATGGCTCCACTCAGATCTCTACACACTCAGCCACATCGAGGCCGAGCTCGGCGCGAACATGCCACCGCCGCGCTGGCGTCAGCAGACCACGTACAAAGCGGCTCCGACGCCGCTAGGGCGGAATTGCGCACTGTTCGATTCCGTCAGGTTGTGGGCCTATCTTCCCGCCCTCATGCGGATCTACCTGCCGACCCGGAACGTGGACGGACTCGGCCGCGCGATCTATGCCGAGTGCCACGCGCGAAACGCCGAATTTCCGTGCAACGACGTGTGTCCCGGACCGCTACCGGACAGCGAGGTCCGCGCCATCGCCAACAGCATTTGGCGTTGGATCACAACCAAGTCGCGCATTTGGGCGGACGGGATCGTGGTCTACGAGGCCACACTCAGTGCGCGCCATGCGGCCATCTCGCGGAAGGGCGCAGCAGCGCGCACGGCGGCGAGCACAGTTGCGCGGCGCGCAAAGTCCGCGTCAGCCATGGAGGCATTGCTATGAGCGACGGCTACAGCGACGGCTACAGCGACGGCTACAACTGGCAGCCGACTGTCCGCAAAAAGCGGCGCGTGACCGCCGCCGAAGGCGCTCGAATCACCGGACTATCCGAACGCCACGTCGTCCGGCTCGTGGCGCAGGAACGCAGCGAGTGGTTCGCCGAGCAGGCTGCACGCCGCGAACGCATCCGCGCCTATCACGACGACGAGGGCCACTCTTGGCCGCAAACGGCCAAACATTTCGGGCTGCATCTGGACACCGTTAAGCGACTCGGCTATCGGGCGAGGAAAGAGCGTGCGGCAGAACAGGAAGCGGCTCAAAAGGCCCACAACGAAGCCGACAATCCACCGCTGTTCTAACGCAATTGGGGAGCGGGTGTCGCGGGGGTTCCGTGGGGGGTTCCGTTGCAACGGGTCGGACAGGTAAAAGTCCTGGTAGACGCTAGTTTTCTGGTTTGGGCCATGCCTGTCTCGTTGCGTGTTTCGTTGCGTCCGTTTTGAATACCAGCCAGACGAGACGGGGTTCTACGAATCTTGGTCGATACCAAGCCATTTCCGCTGAATATCGTGGAGCTCACCGCCAGAATCGGTGGTTGTGGTGATGTACGTGGCGAACTCCGTTGTAGTGCTTGTGGTGGCATCCGTGGCGCGGCCGCGGTACCAGTGACGCGGTCTCAAGCGTCGAGCGTCGCCAGCGTGTCGAGGATGTCGAAGTCGTAGCCGTCGGCGCTGGCGATGTAGACCTGCTGGTCGAATTGACTGTCGCGCATACACATCGGGCCCCGGGGCCCGTCGAACCCGACATCGTGCGCGGATGCCATCAGGTCCGGTATCTCGGGGGAGTGGGCCCGCTGGAAGATGGCCTCGAGCGCAAGCAGACCCTCGTAACAGGATTCGGCCATCGCGTTGAGCGGTGGCGCGTCGGCGCCGTAGCGGGCGACGTAGCTGCCCATCAGGTCCATGGCACCCGCGGTGGCCAGTGAACTGAAGTACGCCGCGGCGACATAGAGGTTTTCGGTGGAGCCGGCGCCGCTGGCCAGCAGCATGTTCTCCTCCATCAGCGGGCTGAACCGCGCCATGCGGTCGTGCCCGCCGGCGCGCGCGAACTCGCGGTTGAACAACACGGCGTCCTGGCCGACGAGCAGCATCAACACGGCCTGCGCCCCCGACGCGATGGCCTTGCGGACAGGTGCGCGGAAATCGTCGGTGCCGTACGGGACGTAGATCTCCCGTCTGAGCTCGAGGTCCAGATCTCGGCAGTACGCGCGGGCGGCCGCGGCGGAACGGCGCGGCCAGATGTAGTCATCGCCGACCAGGCACCAGGACCGGATGCCGAAGTGGTCGCGCAGCCAGGCGAGCGCGGGCGCGATCTGGATCTGCGGTGTCTCGCCTGTGCAGAACACGCCCGGTGTGCGTTCACCGCCCTCGTACAACGAGGTGTAGACGTACGGGATGCGGTCGCGGACCACCGGGGAGATGCGGTTGCGCACGGCCGAGATGTGCCAGCCGGTCACGGCGTCGAGACCGTGACCTCGCAACCGGTCGGCGACGGTCCGGGCGACGTCGTCGCCGGGCGCTCCGCCGTCGAGCACCTCGATGGTGACCTTGCGGCCCTGCAGGCCGCCTCGGTCGTTGACCTCCTTGGCCGCGAGCTCGGCCACGGCCTCGCACGAAGGCGCGAAGATTCCCGCTGGCCCTTGAAGCGGAATCACCAGCCCGACGCGGAACTCAACCTCGCCGTCCTGCACTCCAGATCACCGTCGATCCCGTGTAGTCTGCGCTTCAAAGCTTTCTAGCAGAAATAATTCATTCTGAACAGACCCCGCCGTCGACACGAGGAGACACCCACCATGGCCGCCGGACAGCAGCGCCGCCCCAACCTCCTGCTGCCGTTGGTGCGTCTGACCCACCTCGCGGAGTCGGCGATCGAACGCGTGCTCGCGGACTCGTCGCTCAAGATCGAGGACTGGCGCGTGCTCGACGAGTTGGCCGGACGGCGCACCGTGCCCATGAGCGATCTCGCGCAGGCCACGCTGATCACGGGTCCGACTCTCACCAGAACCGTCGATCGCCTTGTGTCGCAAGGGATCATCTACCGGACTGCCGATCTGCATGACCGCCGGCGGGTGCTCGTGGCGTTGACCCCGCGGGGGCGGACGCTGCGCAACCGCCTGGTGGACGCGGTAGCCGAGGCCGAGTGTGCGGCTTTTGAATCGTGCGGGCTGGACGTCGACCAGTTGCGCGAACTCGTCGACACCACCTCGAATTTGACTTCGTAACCACCCGCGCCCGGCGCGGGCGTTCACCCTTGACTTTTATTTTCATCTGGATATATTTCGGGTGAATGGAAAGGGGTGACCATGCCGACCTACACATTCCGTTGTTCCCACTGCGGTCCCTTCGATCTCACCTGCGCGATCTCCGAGCGCGATGCGGCGGCGACCTGTCCGGAGTGCCGGACGCCGGCGCGCCGGGTCTTCGGTTCGGTAGGGCTGACGACATTCACCGCGGGACATCACCGCGCATTCGACGCGGCGTCCGCGAGCGCCGAAAGTCCCACGGTGGTGAAGTCGATTCCCGCAGGCGCGGACCGCCCGCGGGCCCCGCGCCGCAATCCCGGTCTACCGAGTCTGCCGAGGTACTAGCGACATGGGTGGCGTCGGGCTCTTCTACGTGGGTGCGGTGCTCATCATCGACGGGCTGATGCTGCTGGGCCGCATCAGCCCACGAGGCGCAACACCGCTGAACTTCTTCGTCGGCGGACTGCAGGTGGTGACGCCTACGGTGCTGATCCTGCAGTCCGGCGGAGACGCGGCCGTGATCTTCGCGGCCTCCGGGCTCTACCTGTTCGGCTTCACCTACCTGTGGGTGGCCATCAACAACGTGACCGACTGGGACGGAGAAGGTCTCGGATGGTTCTCGCTGTTCGTCGCGATCGCCGCACTCGGCTACTCGTGGCACGCGTTCACCGCCGAGGCCGACCCGGCGTTCGGGGTGATCTGGCTGCTGTGGGCAGTGCTGTGGTTCATGCTGTTCCTGCTGCTCGGCCTGGGGCACGACGCACTGGGGCCCGCCGTCGGGTTCGTCGCGGTGGCCGAAGGCGTGATCACCGCCGCCGTGCCGGCCTTCCTGATCGTGTCGGGCAACTGGGAAACCGGCCCGCTCCCCGCCGCGGTCATCGCCGTGATCGGTTTTGCCGCAGTTGTTCTCGCATACCCCATCGGGCGCCGTCTCGCAGCGCCGTCAGTCACCAACCCTCCACCGGCCGCGCTCGCGGCCACCACCCGATAAGAGAAAGGGAGTCCACATGCCCGAGGTAGTTTTGGATCCT**CACCACCACCACCACCAC**ACTAGTCAGCTGCAGAATTCATATGCATCGATGGT**TAA**CTAGCGTACGATCGACTGCCAGGCATCAAATAAAACGAAAGGCTCAGTCGAAAGACTGGGCCTTTCGTTTTATGCCATCATGGCCGCGGTGATCAGCTAGCCACCTGACGTCGGGGGGGGGGG

The 6×His tag site is indicated in red. The termination codon site is indicated in bold.


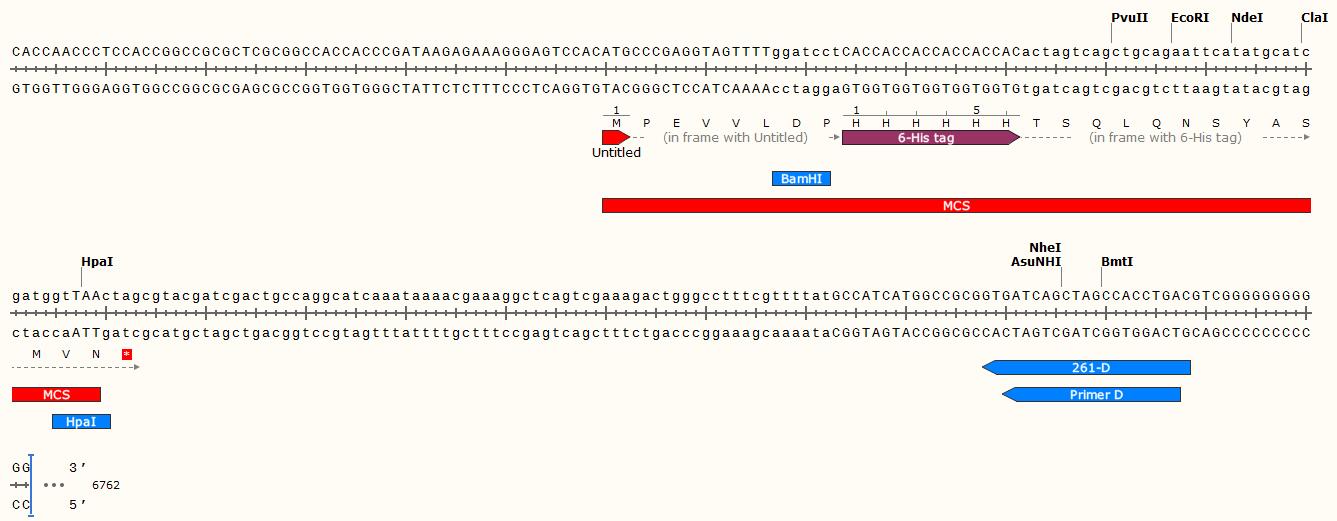


1. **pMV361-eGFP sequence and eGFP site**

AATGTAGTCTTATGCAATACTCTTGTAGTCTTGCAACATGGTAACGATGAGTTAGCAACATGCCTTACAAGGAGAGAAAAAGCACCGTGCATGCCGATTGGTGGAAGTAAGGTGGTACGATCGTGCCTTATTAGGAAGGCAACAGACGGGTCTGACATGGATTGGACGAACCACTGAATTGCCGCATTGCAGAGATATTGTATTTAAGTGCCTAGCTCGATACATAAACGGGTCTCTCTGGTTAGACCAGATCTGAGCCTGGGAGCTCTCTGGCTAACTAGGGAACCCACTGCTTAAGCCTCAATAAAGCTTGCCTTGAGTGCTTCAAGTAGTGTGTGCCCGTCTGTTGTGTGACTCTGGTAACTAGAGATCCCTCAGACCCTTTTAGTCAGTGTGGAAAATCTCTAGCAGTGGCGCCCGAACAGGGACTTGAAAGCGAAAGGGAAACCAGAGGAGCTCTCTCGACGCAGGACTCGGCTTGCTGAAGCGCGCACGGCAAGAGGCGAGGGGCGGCGACTGGTGAGTACGCCAAAAATTTTGACTAGCGGAGGCTAGAAGGAGAGAGATGGGTGCGAGAGCGTCAGTATTAAGCGGGGGAGAATTAGATCGCGATGGGAAAAAATTCGGTTAAGGCCAGGGGGAAAGAAAAAATATAAATTAAAACATATAGTATGGGCAAGCAGGGAGCTAGAACGATTCGCAGTTAATCCTGGCCTGTTAGAAACATCAGAAGGCTGTAGACAAATACTGGGACAGCTACAACCATCCCTTCAGACAGGATCAGAAGAACTTAGATCATTATATAATACAGTAGCAACCCTCTATTGTGTGCATCAAAGGATAGAGATAAAAGACACCAAGGAAGCTTTAGACAAGATAGAGGAAGAGCAAAACAAAAGTAAGACCACCGCACAGCAAGCGGCCGCTGATCTTCAGACCTGGAGGAGGAGATATGAGGGACAATTGGAGAAGTGAATTATATAAATATAAAGTAGTAAAAATTGAACCATTAGGAGTAGCACCCACCAAGGCAAAGAGAAGAGTGGTGCAGAGAGAAAAAAGAGCAGTGGGAATAGGAGCTTTGTTCCTTGGGTTCTTGGGAGCAGCAGGAAGCACTATGGGCGCAGCGTCAATGACGCTGACGGTACAGGCCAGACAATTATTGTCTGGTATAGTGCAGCAGCAGAACAATTTGCTGAGGGCTATTGAGGCGCAACAGCATCTGTTGCAACTCACAGTCTGGGGCATCAAGCAGCTCCAGGCAAGAATCCTGGCTGTGGAAAGATACCTAAAGGATCAACAGCTCCTGGGGATTTGGGGTTGCTCTGGAAAACTCATTTGCACCACTGCTGTGCCTTGGAATGCTAGTTGGAGTAATAAATCTCTGGAACAGATTTGGAATCACACGACCTGGATGGAGTGGGACAGAGAAATTAACAATTACACAAGCTTAATACACTCCTTAATTGAAGAATCGCAAAACCAGCAAGAAAAGAATGAACAAGAATTATTGGAATTAGATAAATGGGCAAGTTTGTGGAATTGGTTTAACATAACAAATTGGCTGTGGTATATAAAATTATTCATAATGATAGTAGGAGGCTTGGTAGGTTTAAGAATAGTTTTTGCTGTACTTTCTATAGTGAATAGAGTTAGGCAGGGATATTCACCATTATCGTTTCAGACCCACCTCCCAACCCCGAGGGGACCCGACAGGCCCGAAGGAATAGAAGAAGAAGGTGGAGAGAGAGACAGAGACAGATCCATTCGATTAGTGAACGGATCTCGACGGTATCGGTTTTAAAAGAAAAGGGGGGATTGGGGGGTACAGTGCAGGGGAAAGAATAGTAGACATAATAGCAACAGACATACAAACTAAAGAATTACAAAAACAAATTACAAAAATTCAAAATTTTATCGACCTGAGGTAATTATAACCCGGGCCCTATATATGGATCCAATTGCAATGATCATCATGACAGATCTGCGCGCGATCGATCTTACGCGTGCTAGCCCAATTCGATAATTCCACGGGGTTGGGGTTGCGCCTTTTCCAAGGCAGCCCTGGGTTTGCGCAGGGACGCGGCTGCTCTGGGCGTGGTTCCGGGAAACGCAGCGGCGCCGACCCTGGGTCTCGCACATTCTTCACGTCCGTTCGCAGCGTCACCCGGATCTTCGCCGCTACCCTTGTGGGCCCCCCGGCGACGCTTCCTGCTCCGCCCCTAAGTCGGGAAGGTTCCTTGCGGTTCGCGGCGTGCCGGACGTGACAAACGGAAGCCGCACGTCTCACTAGTACCCTCGCAGACGGACAGCGCCAGGGAGCAATGGCAGCGCGCCGACCGCGATGGGCTGTGGCCAATAGCGGCTGCTCAGCAGGGCGCGCCGAGAGCAGCGGCCGGGAAGGGGCGGTGCGGGAGGCGGGGTGTGGGGCGGTAGTGTGGGCCCTGTTCCTGCCCGCGCGGTGTTCCGCATTCTGCAAGCCTCCGGAGCGCACGTCGGCAGTCGGCTCCCTCGTTCCGAATTGGGCTCGACCGGTCGCCACC**ATGGTGAGCAAGGGCGAGGAGCTGTTCACCGGGGTGGTGCCCATCCTGGTCGAGCTGGACGGCGACGTAAACGGCCACAAGTTCAGCGTGTCCGGCGAGGGCGAGGGCGATGCCACCTACGGCAAGCTGACCCTGAAGTTCATCTGCACCACCGGCAAGCTGCCCGTGCCCTGGCCCACCCTCGTGACCACCCTGACCTACGGCGTGCAGTGCTTCAGCCGCTACCCCGACCACATGAAGCAGCACGACTTCTTCAAGTCCGCCATGCCCGAAGGCTACGTCCAGGAGCGCACCATCTTCTTCAAGGACGACGGCAACTACAAGACCCGCGCCGAGGTGAAGTTCGAGGGCGACACCCTGGTGAACCGCATCGAGCTGAAGGGCATCGACTTCAAGGAGGACGGCAACATCCTGGGGCACAAGCTGGAGTACAACTACAACAGCCACAACGTCTATATCATGGCCGACAAGCAGAAGAACGGCATCAAGGTGAACTTCAAGATCCGCCACAACATCGAGGACGGCAGCGTGCAGCTCGCCGACCACTACCAGCAGAACACCCCCATCGGCGACGGCCCCGTGCTGCTGCCCGACAACCACTACCTGAGCACCCAGTCCGCCCTGAGCAAAGACCCCAACGAGAAGCGCGATCACATGGTCCTGCTGGAGTTCGTGACCGCCGCCGGGATCACTCTCGGCATGGACGAGCTGTACAAGTAA**AGCGGCCGCGACTCTAGAGGTACCGGTTGTTAACGTTAGCCGGCTACATGGCGCAATCACTAGAGCGGCCGCCACCGCGGAATTAATTCGAGCTCGGTACCTTTAAGACCAATGACTTACAAGGCAGCTGTAGATCTTAGCCACTTTTTAAAAGAAAAGGGGGGACTGGAAGGGCTAATTCACTCCCAACGAAGACAAGATCTGCTTTTTGCTTGTACTGGGTCTCTCTGGTTAGACCAGATCTGAGCCTGGGAGCTCTCTGGCTAACTAGGGAACCCACTGCTTAAGCCTCAATAAAGCTTGCCTTGAGTGCTTCAAGTAGTGTGTGCCCGTCTGTTGTGTGACTCTGGTAACTAGAGATCCCTCAGACCCTTTTAGTCAGTGTGGAAAATCTCTAGCAGTAGTAGTTCATGTCATCTTATTATTCAGTATTTATAACTTGCAAAGAAATGAATATCAGAGAGTGAGAGGAACTTGTTTATTGCAGCTTATAATGGTTACAAATAAAGCAATAGCATCACAAATTTCACAAATAAAGCATTTTTTTCACTGCATTCTAGTTGTGGTTTGTCCAAACTCATCAATGTATCTTATCATGTCTGGCTCTAGCTATCCCGCCCCTAACTCCGCCCATCCCGCCCCTAACTCCGCCCAGTTCCGCCCATTCTCCGCCCCATGGCTGACTAATTTTTTTTATTTATGCAGAGGCCGAGGCCGCCTCGGCCTCTGAGCTATTCCAGAAGTAGTGAGGAGGCTTTTTTGGAGGCCTAGGGACGTACCCAATTCGCCCTATAGTGAGTCGTATTACGCGCGCTCACTGGCCGTCGTTTTACAACGTCGTGACTGGGAAAACCCTGGCGTTACCCAACTTAATCGCCTTGCAGCACATCCCCCTTTCGCCAGCTGGCGTAATAGCGAAGAGGCCCGCACCGATCGCCCTTCCCAACAGTTGCGCAGCCTGAATGGCGAATGGCGCGACGCGCCCTGTAGCGGCGCATTAAGCGCGGCGGGTGTGGTGGTTACGCGCAGCGTGACCGCTACACTTGCCAGCGCCCTAGCGCCCGCTCCTTTCGCTTTCTTCCCTTCCTTTCTCGCCACGTTCGCCGGCTTTCCCCGTCAAGCTCTAAATCGGGGGCTCCCTTTAGGGTTCCGATTTAGTGCTTTACGGCACCTCGACCCCAAAAAACTTGATTAGGGTGATGGTTCACGTAGTGGGCCATCGCCCTGATAGACGGTTTTTCGCCCTTTGACGTTGGAGTCCACGTTCTTTAATAGTGGACTCTTGTTCCAAACTGGAACAACACTCAACCCTATCTCGGTCTATTCTTTTGATTTATAAGGGATTTTGCCGATTTCGGCCTATTGGTTAAAAAATGAGCTGATTTAACAAAAATTTAACGCGAATTTTAACAAAATATTAACGTTTACAATTTCCCAGGTGGCACTTTTCGGGGAAATGTGCGCGGAACCCCTATTTGTTTATTTTTCTAAATACATTCAAATATGTATCCGCTCATGAGACAATAACCCTGATAAATGCTTCAATAATATTGAAAAAGGAAGAGTATGAGTATTCAACATTTCCGTGTCGCCCTTATTCCCTTTTTTGCGGCATTTTGCCTTCCTGTTTTTGCTCACCCAGAAACGCTGGTGAAAGTAAAAGATGCTGAAGATCAGTTGGGTGCACGAGTGGGTTACATCGAACTGGATCTCAACAGCGGTAAGATCCTTGAGAGTTTTCGCCCCGAAGAACGTTTTCCAATGATGAGCACTTTTAAAGTTCTGCTATGTGGCGCGGTATTATCCCGTATTGACGCCGGGCAAGAGCAACTCGGTCGCCGCATACACTATTCTCAGAATGACTTGGTTGAGTACTCACCAGTCACAGAAAAGCATCTTACGGATGGCATGACAGTAAGAGAATTATGCAGTGCTGCCATAACCATGAGTGATAACACTGCGGCCAACTTACTTCTGACAACGATCGGAGGACCGAAGGAGCTAACCGCTTTTTTGCACAACATGGGGGATCATGTAACTCGCCTTGATCGTTGGGAACCGGAGCTGAATGAAGCCATACCAAACGACGAGCGTGACACCACGATGCCTGTAGCAATGGCAACAACGTTGCGCAAACTATTAACTGGCGAACTACTTACTCTAGCTTCCCGGCAACAATTAATAGACTGGATGGAGGCGGATAAAGTTGCAGGACCACTTCTGCGCTCGGCCCTTCCGGCTGGCTGGTTTATTGCTGATAAATCTGGAGCCGGTGAGCGTGGGTCTCGCGGTATCATTGCAGCACTGGGGCCAGATGGTAAGCCCTCCCGTATCGTAGTTATCTACACGACGGGGAGTCAGGCAACTATGGATGAACGAAATAGACAGATCGCTGAGATAGGTGCCTCACTGATTAAGCATTGGTAACTGTCAGACCAAGTTTACTCATATATACTTTAGATTGATTTAAAACTTCATTTTTAATTTAAAAGGATCTAGGTGAAGATCCTTTTTGATAATCTCATGACCAAAATCCCTTAACGTGAGTTTTCGTTCCACTGAGCGTCAGACCCCGTAGAAAAGATCAAAGGATCTTCTTGAGATCCTTTTTTTCTGCGCGTAATCTGCTGCTTGCAAACAAAAAAACCACCGCTACCAGCGGTGGTTTGTTTGCCGGATCAAGAGCTACCAACTCTTTTTCCGAAGGTAACTGGCTTCAGCAGAGCGCAGATACCAAATACTGTCCTTCTAGTGTAGCCGTAGTTAGGCCACCACTTCAAGAACTCTGTAGCACCGCCTACATACCTCGCTCTGCTAATCCTGTTACCAGTGGCTGCTGCCAGTGGCGATAAGTCGTGTCTTACCGGGTTGGACTCAAGACGATAGTTACCGGATAAGGCGCAGCGGTCGGGCTGAACGGGGGGTTCGTGCACACAGCCCAGCTTGGAGCGAACGACCTACACCGAACTGAGATACCTACAGCGTGAGCTATGAGAAAGCGCCACGCTTCCCGAAGGGAGAAAGGCGGACAGGTATCCGGTAAGCGGCAGGGTCGGAACAGGAGAGCGCACGAGGGAGCTTCCAGGGGGAAACGCCTGGTATCTTTATAGTCCTGTCGGGTTTCGCCACCTCTGACTTGAGCGTCGATTTTTGTGATGCTCGTCAGGGGGGCGGAGCCTATGGAAAAACGCCAGCAACGCGGCCTTTTTACGGTTCCTGGCCTTTTGCTGGCCTTTTGCTCACATGTTCTTTCCTGCGTTATCCCCTGATTCTGTGGATAACCGTATTACCGCCTTTGAGTGAGCTGATACCGCTCGCCGCAGCCGAACGACCGAGCGCAGCGAGTCAGTGAGCGAGGAAGCGGAAGAGCGCCCAATACGCAAACCGCCTCTCCCCGCGCGTTGGCCGATTCATTAATGCAGCTGGCACGACAGGTTTCCCGACTGGAAAGCGGGCAGTGAGCGCAACGCAATTAATGTGAGTTAGCTCACTCATTAGGCACCCCAGGGCTTTACACTTTATGCTTCCGGCTCGTATGTTGTGTGGAATTGTGAGCGGATAACAATTTCACACAGGAAACAGCTATGACCATGATTACGCCAAGCGCGCAATTAACCCTCACTAAAGGGAACAAAAGCTGGAGCTGCAAGCTT

The eGFP tag site is indicated in green.


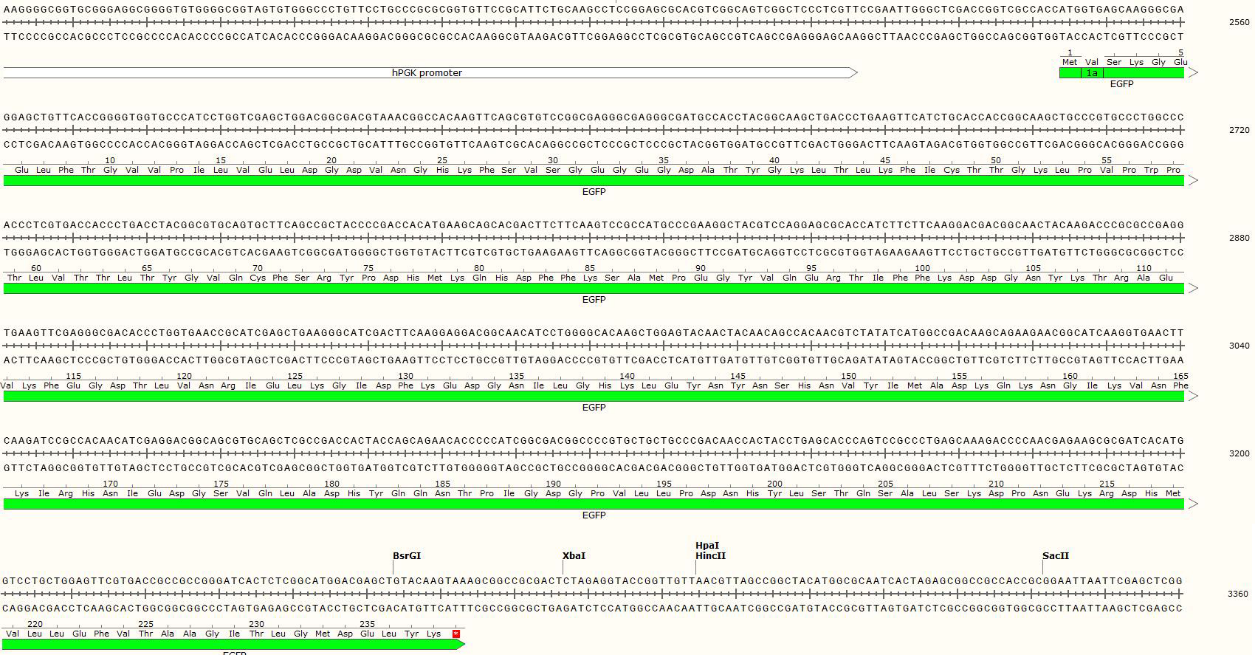

Supplement: Supplementary file 1 [file Data_Sheet_1.docx]
